# Dairy Intake Enhances Body Weight and Composition Changes during Energy Restriction in 18–50-Year-Old Adults—A Meta-Analysis of Randomized Controlled Trials

**DOI:** 10.3390/nu8070394

**Published:** 2016-07-01

**Authors:** Welma Stonehouse, Thomas Wycherley, Natalie Luscombe-Marsh, Pennie Taylor, Grant Brinkworth, Malcolm Riley

**Affiliations:** 1 Commonwealth Scientific Industrial Research Organisation, Adelaide 5000, South Australia, Australia; natalie.luscombe-marsh@csiro.au (N.L.-M.); pennie.taylor@csiro.au (P.T.); grant.brinkworth@csiro.au (G.B.); malcolm.riley@csiro.au (M.R.); 2 School of Health Sciences, University of South Australia, Adelaide 5000, South Australia, Australia; tom.wycherley@unisa.edu.au

**Keywords:** dairy, dairy supplements, body weight, body fat mass, body lean mass, body composition, energy restriction

## Abstract

Background/Aims: A meta-analysis of randomized controlled trials (RCTs) was performed to investigate the effects of dairy food or supplements during energy restriction on body weight and composition in 18–50-year-old. Methods: RCTs ≥ 4 weeks comparing the effect of dairy consumption (whole food or supplements) with control diets lower in dairy during energy restriction on body weight, fat and lean mass were identified by searching MEDLINE, EMBASE, Pubmed, Cochrane Central and World Health Organization International Clinical Trials Registry Platform (WHO ICTRP) until March 2016. Reports were identified and critically appraised in duplicate. Data were pooled using random-effects meta-analysis. Chi^2^- and *I*^2^-statistics indicated heterogeneity. Dose effect was assessed using meta-regression analysis. GRADE guidelines were used to rate the quality (QR) of the evidence considering risk of bias, inconsistency, indirectness, imprecision, publication bias and effect estimates. Results: 27 RCTs were reviewed. Participants consumed between 2 and 4 standard servings/day of dairy food or 20–84 g/day of whey protein compared to low dairy control diets, over a median of 16 weeks. A greater reduction in body weight (−1.16 kg [−1.66, −0.66 kg], *p* < 0.001, *I*^2^ = 11%, QR = high, *n* = 644) and body fat mass (−1.49 kg [−2.06, −0.92 kg], *p* < 0.001, *I*^2^ = 21%, *n* = 521, QR = high) were found in studies largely including women (90% women). These effects were absent in studies that imposed resistance training (QR = low-moderate). Dairy intake resulted in smaller loss of lean mass (all trials pooled: 0.36 kg [0.01, 0.71 kg], *p* = 0.04, *I*^2^ = 64%, *n* = 651, QR = moderate). No between study dose-response effects were seen. Conclusions: Increased dairy intake as part of energy restricted diets resulted in greater loss in bodyweight and fat mass while attenuating lean mass loss in 18–50-year-old adults. Further research in males is needed to investigate sex effects.

## 1. Introduction

Obesity is a global epidemic, with significant health and socio-economic costs [1]. Overweight and obesity are major risk factors for non-communicable diseases such as cardiovascular disease, diabetes, musculoskeletal disorders and some cancers [1]. Hence, the critical necessity to identify effective strategies to reduce and maintain a healthy body weight.

Dairy in the diet may be an important modifiable factor for managing a healthy body weight and composition due to its rich source of nutrients and bioactive compounds. Specifically, dairy contains calcium which may increase faecal fat excretion [2]; protein (casein and whey) and their peptide derivatives that promote muscle protein synthesis (hypertrophy) [3] and regulate appetite [4]; and fatty acids (medium-chain triacylglycerols (MCT) and conjugated linoleic acid (CLA)) that affect energy balance through reduced de novo lipogenesis, increased fat oxidation and regulating appetite [4,5]. Evidence from observational studies suggests a protective effect of dairy food consumption on overweight and obesity risk [6].

Lifestyle modification, incorporating an energy-reduced diet and increased physical activity, is the cornerstone for achieving and maintaining a healthy body weight and composition. However, energy restricted diets aimed at reducing body weight and fat mass often result in a concurrent unfavorable lean mass reduction, accounting for ~20% of total weight loss [7]. Lean mass, particularly its skeletal muscle component, is important for regulation of resting energy expenditure, protein metabolism, physical strength and function and is the body’s primary site of postprandial glucose uptake [8]. Effective weight loss strategies should therefore aim to reduce body weight by reducing body fat mass whilst minimizing lean mass loss. Hence, identifying effective strategies that can promote healthy body weight and composition is critical to combat the overweight/obesity epidemic.

Two previous meta-analyses have reported modest benefits of dairy food in adults ≥18 years on body weight and composition in randomized controlled trials (RCTs) imposing energy restriction [9,10]. Although Abargouei et al. [10] reported lean mass as an outcome, Chen et al. [9] which was a more comprehensive meta-analysis, did not. Furthermore, neither meta-analysis considered dairy supplements nor attempted to determine the dose of dairy required to improve body weight and composition. 

The primary aim of this study was to conduct a systematic literature review and meta-analysis of RCTs in 18–50-year-old investigating the effects of consuming whole dairy food or dairy protein supplements (whey and/or casein) compared to a control intervention lower in dairy products during energy restriction on body weight and composition (total fat mass and lean mass). Secondary aims were to determine the critical dairy dose needed to achieve changes in body weight and composition and to perform a priori subgroup analysis to investigate the influence of the following study characteristics on the outcomes: addition of resistance training vs. no resistance training; whole dairy food vs. dairy supplements; study duration <12 months vs. ≥12 months; the influence of sex; and baseline dairy intake. 

If improved weight loss and body composition due to an increased intake (or particular dose) of dairy products are confirmed through this meta-analysis of RCT it will provide the highest level of scientific evidence to support recommendations and health claims for incorporating dairy products into weight management programs. The evidence may also promote the development of innovative food or therapeutic products for this purpose. Additionally, the review may identify gaps in the current evidence that is required to satisfy health claims for the dairy industry. 

## 2. Materials and Methods

### 2.1. Eligibility Criteria

Relevant published and unpublished studies were included if: they used an RCT study design; were conducted in adults 18–50 years (the upper limit of the age range was flexible and studies reporting an average age of ≤50 years were also included); used interventions of ≥4 weeks [8,9] that primarily involved dairy products (whole dairy foods or dairy supplements) with comparison to an appropriate control diet lower in dairy products; while prescribing energy restriction; and included measures of body weight, fat mass and/or lean mass. Studies were excluded if: they involved multi-component interventions where the effect of dairy could not be distinguished from other components (e.g., another dietary factor added to the dairy, but not to the control, intervention; if the same additional component was added to both dairy and control interventions (e.g., exercise training), the research report was included); used a cross-over study design that do not permit return to equal baseline following body weight/composition changes; used another dairy product or component as comparison (e.g., whey vs. casein); included participants with a disease condition associated with muscle wasting (e.g., human immunodeficiency virus (HIV), cancer, muscular dystrophy); included participants who were pregnant or breastfeeding; or were not available in English language. No exclusions were made based on sex, other pre-existing disease or physiological conditions (e.g., diabetes, cardiovascular disease, polycystic ovary syndrome (PCOS), or metabolic syndrome).

Whole dairy foods were defined as including milk, yoghurt, cheese and custard and serving sizes were based on the Australian Guide to Healthy Eating [11], namely: 250 mL milk and milk-based beverages; 200 g yoghurt; 40 g hard, firm, soft and low fat cheese; 120 g cottage and ricotta cheese; 200 g custard; and 30 g powdered milk. Dairy supplements were defined as including whey and/or casein protein in the form of whey and/or casein concentrates, isolates or hydrolysates. No relevant studies using any other form of dairy supplements were identified. 

### 2.2. Literature Search

Relevant original studies published to 22 March 2016 (first search conducted on 2 June 2014 and updated on 22 March 2016) were identified by a comprehensive systematic search of scientific journal databases (MEDLINE on Web of Science, EMBASE, Pubmed, Cochrane Central and World Health Organization International Clinical Trials Registry Platform (WHO ICTRP) and scanning reference lists of included reports, key meta-analyses and reviews. Two search themes were specified based on the dairy intervention and outcome variables using relevant medical subject heading (MeSH) terms and key words. Dairy intervention search theme: The MeSH term dairy products captured the terms whey, casein, milk, yoghurt (different spellings) and cheese; the following key words not captured by the MeSH term were added: dairy bioactive (different spellings) and dairy calcium. Body Composition: The MeSH term “body composition” was combined with key words weight loss, weight reduction, weight change, fat mass, fat free mass (different spellings) and lean mass. The two themes were then combined and further filtered by adults, English language, clinical trial and human. The following is an example of the search syntax used for searching Pubmed: (((((“Dairy Products” (Mesh) NOT margarine NOT “infant formula” NOT “human milk”)) OR (“dairy bioactive *” OR “dairy bio-active *” OR “dairy calcium”))) AND (“Body Composition” (Mesh) OR “weight loss” OR “weight reduction” OR “weight change” OR “fat mass” OR “fat free mass” OR “fat-free mass” OR “lean mass”)) AND adult Filters: Clinical Trial; Humans.

### 2.3. Study Selection

All duplicate articles were removed. Relevant reports were identified in duplicate by two independent investigators (PT, Paul Foster (PF)) by first screening the titles and abstracts followed by the full text against inclusion and exclusion criteria. Any disagreement was resolved by consensus with a third investigator (WS) experienced in the field of nutrition interventions and body weight regulation. 

### 2.4. Data Extraction and Quality Assessment

Detailed information needed for an in-depth assessment of the evidence were extracted from the studies into pre-piloted extraction tables by two investigators (PT, PF). A third investigator (WS) scrutinized the extractions for errors or inconsistencies. When necessary, errors or uncertainties were resolved by discussion between investigators. The following critical information were extracted: first author’s family name; year of publication; geographic location; primary objective; funding source and conflict of interest; study design (duration, randomization procedures, blinding, treatment allocation concealment); participant information (sample size (numbers enrolled and completed), attrition and reasons, sex distribution, mean age, mean body mass index (BMI), inclusion and exclusion criteria); potential confounding factors that was not controlled for was identified; details regarding intervention and control regimes; mean dietary intakes (dairy, energy, macronutrients, calcium); exercise protocol (when applicable); methods used to assess outcomes; outcome results; author’s conclusions and contact details. The mean and standard deviation (SD) at baseline, end and change for body weight, fat mass and lean mass were extracted. Fat free mass was extracted when lean mass was not available. When multiple time points were reported only the end of intervention point was used. Since most studies reported completer’s data and not intention-to-treat, data for completers were extracted except in a few cases where only complier or intention-to-treat data was available. In cases where there were multiple reports of the same study, the data were extracted from the most relevant report. One report included more than one dairy intervention arm to investigate different dosages. In this case the data were extracted as two separate comparisons (i.e., moderate dairy dose vs. control and high dairy dose vs. control) [12].

A critical appraisal of studies was undertaken in duplicate by two investigators (PT, PF) using the Health Canada Quality appraisal tool for intervention studies [13]. Disagreements were resolved by consensus with a third investigator (WS) experienced in the field of nutrition interventions and weight regulation. A quality score of ≤7 was considered lower quality [13]. No studies were excluded based on quality score, but sensitivity analysis was conducted to assess the impact of these studies on the overall result.

### 2.5. Rating of the Quality of the Body of Evidence

The overall quality of the body of evidence was rated using the Grading of Recommendations Assessment, Development and Evaluation (GRADE) guidelines taking into account the risk of bias, inconsistency, indirectness, imprecision, publication bias and assigning a quality level (high, moderate, low and very low) that reflects confidence that the estimates of the effect are correct [14,15,16,17,18].

### 2.6. Statistical Analysis

A meta-analysis was conducted using Review Manager (RevMan, version 5.2, The Cochrane Collaboration, London, England, 2012). 

For each outcome, the mean change and SD of change from baseline to endpoint for each intervention group was entered into Review Manager. If the SD was not provided, it was calculated from the standard error (SE) or 95% confidence interval (CI). If only baseline and end data was available the mean change was calculated by deducting the baseline from the end value. The SD was then imputed from a mean correlation coefficient of 0.97 calculated from other studies in the meta-analysis that provided SDs for baseline, end and change values [19]. The correlation coefficient calculated was only applied to studies of ≤9 months duration, because fully reported data was not available for studies of longer duration. SDs were imputed for three studies [20,21,22]. Study authors were contacted for missing data or when results were presented in graphs. When no response was received results presented in graphs were estimated by UN-SCAN-IT software for windows (version 7, Silk Scientific Inc., Orem, UT, USA) or the data was not included in the meta-analysis. Data was unable to be retrieved for two studies [23,24]. For studies with multiple comparisons where one group was used more than once as a comparison group, the sample size of the specific group was divided by the number of times the group was used as comparison to avoid data duplication and provide appropriate weighting for the results. 

The primary meta-analyses compared mean (95% CI) differences (net change in kg) in body weight, fat mass and lean mass between high dairy and control groups. Due to heterogeneity between studies and to avoid false positive results for subgroup analysis, a random-effects model was used to calculate Forest plots with weighted mean differences and 95% CI. Heterogeneity between studies were indicated using a combination of Chi^2^- (*p* < 0.1) and *I*^2^ statistics (*I*^2^ 0%–40% = low; 30%–60% = moderate; 50%–90% = substantial and 75%–100% = considerable heterogeneity) and considering the variation of point estimates and overlap of CIs across studies [17,19]. A priori subgroup differences (resistance training vs. no resistance training) and whole dairy food vs. dairy supplements) were assessed using Chi^2^-statistic with a *p*-value of < 0.05 taken to indicate statistical significance [19]. Subgroup differences between studies using skim/low-fat dairy vs. studies that did not specify dairy type were also explored. Subgroup analysis on study duration and baseline dairy intake could not be performed because of limited number of studies that were >12 months duration or that reported dairy intake at baseline. Subgroup analysis also could not be performed on sex because of the low proportion of men included in studies. 

Meta-regression was used to examine the effects of dairy food dosage on effect size (Metareg [25], Stata SE 12.1, College Station, TX, USA), in a random effects analysis of univariate and multivariate linear models. Analyses assuming quadratic relationships or more general curvature were not performed. No attempt was made to include trials using dairy supplements in the same model as trials using dairy whole foods because of the uncertainty of converting the intervention dose to common units.

Sensitivity analyses were conducted by evaluating the impact of adding or removing reports with different study characteristics. Sensitivity analysis was conducted on trials with high apparent bias, trials with imputed data, trials with intention-to-treat data, trials reporting fat free mass (as opposed to lean mass), trials where the difference in dairy servings between groups was <1 servings/day and trials that included only men. 

Publication bias was examined using funnel plots in which the SE of the studies were plotted against their corresponding effect sizes. 

## 3. Results

### 3.1. Literature Search Results

**Figure 1:**
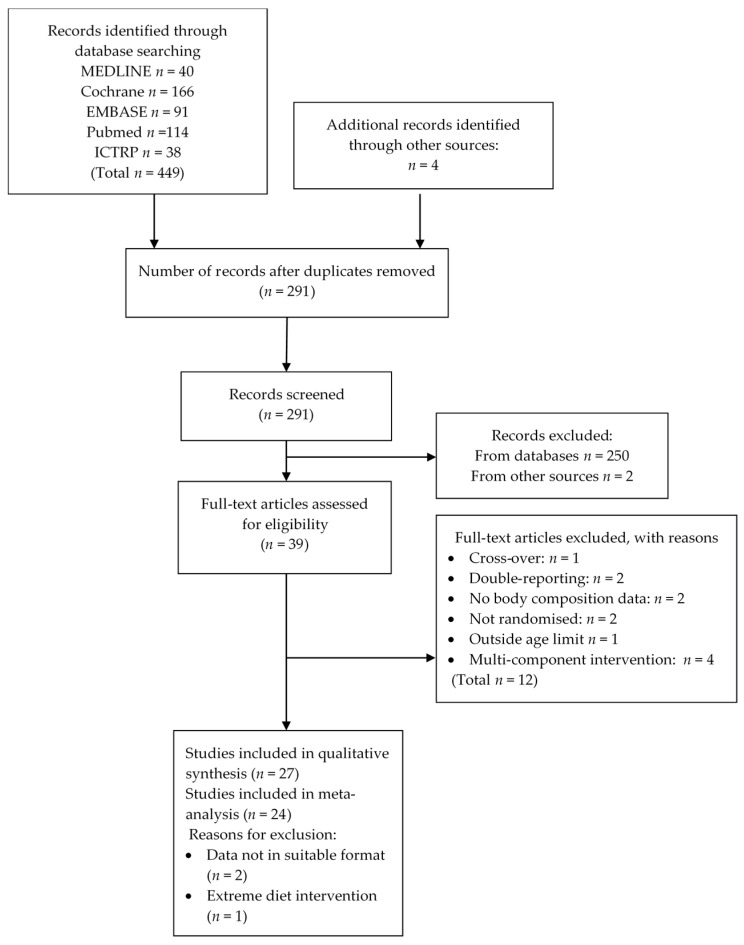
Flow diagram for selection of studies (ICTRP, International Clinical Trials Registry Platform).

The number of studies assessed at each stage of the identification filtering process are summarized in Figure 1. A total of 27 studies satisfied the selection criteria and were included in the systematic literature review equating to a total of 29 comparisons that included 1278 participants (these numbers include all studies including those excluded from the meta-analysis).

Twelve full-text studies were excluded for the following reasons: studies using a cross-over design [26]; double-reporting (sub-studies of other RCT) [27,28]; no body weight or composition data reported [29,30]; interventions not randomized [31,32]; study population outside of age limit [33]; and multi-component interventions [34,35,36,37]. 

Two studies were not included in the meta-analysis [23,24,38] because the critical data was not in a form suitable for standardized extraction and the authors did not respond to information requests. These studies were however included in the qualitative systematic review. Another report was excluded because it used an extreme dietary intervention of milk as the only food source for 16 weeks [38]. 

### 3.2. Characteristics of Included Studies

The characteristics of the RCTs included in the systematic review are summarized in Table 1.

#### 3.2.1. Geographical Location

The majority of trials were conducted in the USA (*n* = 18) with others conducted in Canada (*n* = 2), Australia (*n* = 2), Mexico (*n* = 1), UK (*n* = 1), Iran (*n* = 1), Japan (*n* = 1) and Brazil (*n* = 1).

#### 3.2.2. Study Design

Two studies had intervention periods ≥12 months [39,40] while the remaining studies ranged from 8 to 24 weeks (median duration = 16 weeks (16 weeks for whole dairy food; 8 weeks for dairy supplement studies)).

#### 3.2.3. Study Populations

Most studies were conducted on participants aged 18–50 years, while a few studies exceeded the upper range limit (*n* = 13 studies) (Table 1) Average age across studies was 38 years. Healthy overweight/obese was the target population for most studies (*n* = 24) while other studies included overweight/obese participants with metabolic syndrome (*n* = 1) or PCOS (*n* = 1) (See Table 1). Tanaka et al. [41] recruited Japanese men with ≥2 components of the metabolic syndrome of which BMI was one criteria resulting in a sample with 75% overweight/obese participants. Most studies were conducted in women only or contained large proportions of women (overall 80% of participants were women). Two studies were conducted in men only [41,42], while Bowen et al. included a small sub-sample of men [43].

#### 3.2.4. Interventions

A total of 21 and 6 studies used dairy foods and dairy supplements, respectively as the intervention treatments. 

Dairy foods included daily servings of milk (7 studies), mixed dairy foods including milk, yoghurt and cheese (10 studies) and yoghurt only (2 studies). Anderson et al. [24] provided milk-based meal replacements (Slimfast™) and Gilbert et al. [20] used a milk supplement with additional calcium. Some studies (*n* = 8) specified the use of low-fat or skimmed dairy products. Differences in the amount of dairy food between dairy and control interventions for most studies ranged from ~2 to 4 servings/day. In three studies the difference was less than 2 servings/day (0.75 servings/day [44], 1.3 servings/day [41], 1.8 serving/day [40]. Josse et al. [12] compared multiple dosages of dairy intake (low vs. moderate vs. high dairy intake). Most dairy food studies used a low dairy diet as the comparison group, while some used isocaloric placebo-type controls that were either, soy [24,45], rice [20], carbohydrate [44] or gelatin-based [46]. 

Dairy supplement intakes ranged from 20 to 84 g/day (average 51 g/day) of either whey fraction high in leucine [47] or whey protein isolate [21,42,48,49]. Anderson et al. [23] provided casein protein in the form of shakes. Dairy supplement studies predominantly used isocaloric placebos of either carbohydrate (maltodextrin, glucose) [47,49], soy [23] or gelatin-based ([21]. Aldrich et al. [48] was the only supplement study that used a diet-based control (diet including 1.2 servings of dairy/day + calcium supplements to balance calcium intake between groups). Longland et al. [42] used skim and whole milk as the supplement basis with whey and maltodextrin added for dairy and control groups, respectively (See Table 1). 

Few studies reported level of compliance to the interventions, although the majority implemented compliance assessment. Of the studies reporting compliance, >70% compliance was achieved [20,23,24,41,42,50,51,52,53,54].

#### 3.2.5. Dietary Intakes

Daily intake of protein and calcium at baseline are summarized in Table S1. Few studies reported dairy intake at baseline, but based on inclusion criteria of ≤1 serving/day or reported baseline calcium intake <600 mg/day, low dairy intake at baseline was presumed for 11 studies [12,22,41,44,45,47,52,54,55,56,57] while high intakes of >1 serving/day were assumed for other studies because of high baseline calcium intakes reported (>600 mg/day) [20,39,43,50,53]. Several studies, however, did not report dietary intake at baseline (Table S1). 

Most studies reduced energy intake by >2092 kJ/day (>500 kcal/day), while the energy deficit was less in four studies, ranging from 1046 to 1255 kJ/day (250–300 kcal/day) [22,41,44,50] (Table 1). 

Mean daily nutrient composition of the intervention diets are summarized in Table S2. In most whole dairy food studies dairy servings were incorporated into the diet by replacing certain foods to match energy and protein intakes across groups, but calcium intakes were generally higher in the dairy compared to control, groups. For most studies the amount of protein provided by the dairy food intervention diets were relatively standard (17–22 percentage energy (% E)) and similar between dairy and control groups [12,20,22,40,41,44,46,52,54,55,56,57,58]. Bowen et al. [43] and Lukaszuk et al. [45] provided higher protein diets (~32% E and 27% E, respectively), but the protein content was comparable between dairy and control groups while protein intake was significantly greater than the control groups in the studies of Hawley et al. [50] (30 vs. 21% E; 1.3 vs. 0.9 g/kg) and Josse et al. [12] (High dairy diet: 28 vs. 16% E; 1.3 vs. 0.7 g/kg). 

Dairy supplement interventions provided greater protein intakes compared to control interventions (Aldrich et al.: 1.46 vs. 0.78 g/kg [48]; Kasim-Karakas et al.: 1.07 vs. 0.61 g/kg [49]; Longland et al.: 2.4 vs. 1.2 g/kg [42]) while protein intakes were low (<1 g/kg) and comparable to control interventions in the studies by Frestedt et al. [47] and Piccolo et al. [21].

All whole dairy food studies, except Lukaszuk et al. [45], reported calcium intakes that were greater in the dairy groups compared to the control groups (differences ranged between 141 and 1862 mg/day); the lower end of the range reflecting the smaller difference in dairy servings between groups in two studies [41,44]. Lukaszuk et al. [45] used a soy milk comparison with added soy protein to equate the protein content of the two interventions resulting in similar protein and calcium intakes between groups. Calcium intakes in dairy supplement studies, when reported, were similar between groups [47,48,59].

Fat intake was similar between groups for most studies; differing by <5% E between dairy and control groups except for studies by Hawley et al. [50] and Longland et al. [42]. Carbohydrate intake was similar between groups for most studies except for some where carbohydrate intake was >5% E greater in the control groups compared to the dairy group [12,45,48,49,50]. 

#### 3.2.6. Exercise Regimes

Six studies prescribed resistance training as part of the intervention. Frequency of resistance training was 2 times/week [12], 3 times/week [22,44,50,53] and 6 times/week [42]. Two studies included only resistance training [22,44] while others combined it with aerobic training [12,42,50,53] and one study included only aerobic training [40]. Exercise regimes were similar between dairy and control groups. In two studies dairy was consumed proximate to exercise (before and/or after exercise) [42,44].

#### 3.2.7. Outcome Measures

Most studies used dual energy X-ray absorptiometry (DEXA) to assess body composition and five studies used bioelectrical impedance analysis (BIA) [41,45,49,53,55]. DEXA is considered a valid and precise methodology for assessing changes in body composition while BIA is generally thought to be less appropriate to assess small changes in body fat [60]. Majority of studies reported lean mass while three studies reported fat free mass [20,22,45]. Sensitivity analysis showed no impact of body composition methodology or measurement of fat free mass on the overall results. 

Two studies analyzed the data using an intention-to-treat approach [12,51], one study assessed body weight in participants who complied with the treatments only [40] whereas all other studies analyzed the data for participants who completed the interventions. Sensitivity analysis showed no impact of analysis approach on the summary outcomes. 

Sensitivity analysis also showed no impact of studies where SD had been imputed on outcomes [20,21,22]. 

### 3.3. Quality Assessment of Individual Studies

Most studies were of high quality (quality scores ≥ 8) with only two studies scoring 7 [52,55] and none < 7 (Table 1). Sensitivity analysis showed no impact of removing studies with a quality scores < 8 on heterogeneity or the overall results.

The quality criteria failed by most studies were randomization method and allocation concealment mostly due to incomplete reporting. Due to the nature of the interventions most interventions were not blinded. Confounding factors, not controlled for, most prevalent were potential attrition bias because attrition and related reasons across groups were not reported by several studies; several studies did not report nutrient intakes and differences between groups could not be ascertained (Table S3). No conflict of interest that may have affected study outcomes was apparent in any study.

### 3.4. Effects of Dairy Intake on Body Weight and Fat Mass during Energy Restriction

**Figure 2:**
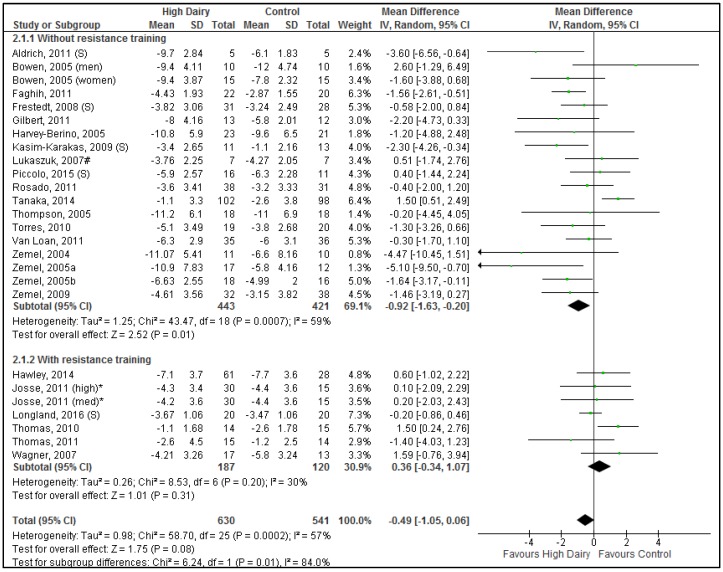
Forrest plot of mean (95% confidence interval (CI)) weighted differences in body weight (kg) between high dairy and control groups stratified for sub-groups with resistance training vs. without resistance training. Results when Tanaka, 2014 [41] excluded: −1.10 kg [−1.65, −0.56 kg], Z = 3.99 (*p* < 0.001); Heterogeneity: Chi^2^ = 21.56, degrees of freedom (*df*) = 17 (*p* = 0.20). (S) The intervention was dairy supplements; # The comparison group was soy protein; * Sample size adapted for multiple comparisons to avoid data duplication; med, medium dairy group; high, high dairy group.

**Figure 3:**
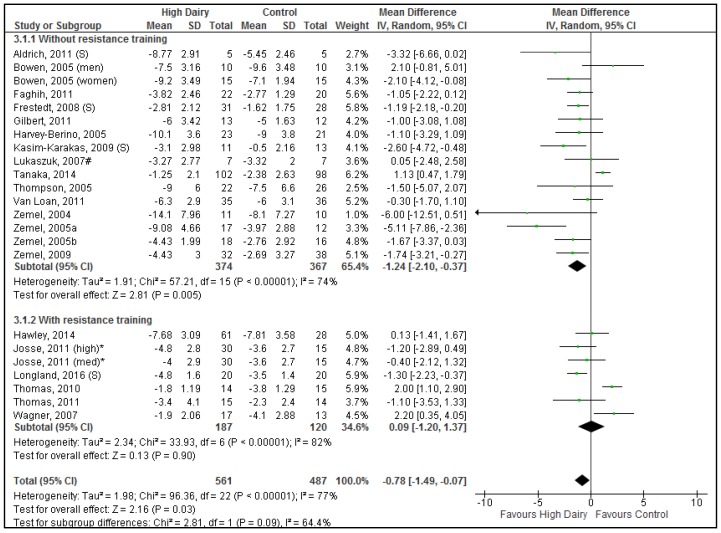
Forrest plot of mean (95% CI) weighted differences in body fat mass (kg) between high dairy and control groups stratified for sub-groups with resistance training vs. without resistance training. Results when Tanaka, 2014 excluded: −1.41 kg [−2.04, −0.77 kg], Z = 4.86 (*p* < 0.001); Heterogeneity: Chi^2^ = 22.61, *df* = 15 (*p* = 0.09). (S) The intervention was dairy supplements; # The comparison group was soy protein; * Sample size adapted for multiple comparisons to avoid data duplication; med, medium dairy group; high, high dairy group.

Significant differences were seen between the no resistance training vs. resistance training subgroups for body weight (Chi^2^ = 6.24, *p* = 0.01) (Figure 2) with a tendency to differ for fat mass (Chi^2^ = 2.98, *p* = 0.08) (Figure 3). Hence results for these subgroups were reported separately.

#### 3.4.1. Energy Restriction without Resistance Training

A total of 864 participants (744 in dairy food studies; 120 in dairy supplement studies) in 19 comparisons (15 using dairy food; 4 using dairy supplement), from 18 studies (14 dairy food; 4 dairy supplements) were included in the meta-analysis for body weight change. For the meta-analysis of body fat mass change, a total of 741 participants (648 in dairy food studies; 93 in dairy supplement studies) in 16 comparisons (13 using dairy food; 3 using dairy supplement, from 15 studies (12 using dairy food; 3 using dairy supplements)) were included. 

Overall (when dairy food and supplement studies were pooled), increased dairy intake combined with energy restriction resulted in a significantly greater reduction in body weight (Figure 2) and body fat mass (Figure 3) compared to control interventions (Body weight: −0.92 kg [−1.63, −0.20 kg], *p* = 0.01; Body fat mass: −1.24 kg [−2.10, −0.37 kg], *p* < 0.01). Results did not differ between dairy food and dairy supplement subgroups (Body weight: Chi^2^ = 0.23, *p* = 0.63; Body fat mass: Chi^2^ = 0.84, *p* = 0.36). Substantial heterogeneity was seen for body weight (*p* < 0.001, *I*^2^ = 59%) and body fat mass (*p* < 0.001, *I*^2^ = 74%). One study had significant impact on heterogeneity; excluding Tanaka, 2014 [41] reduced heterogeneity to acceptable levels for both body weight and body fat mass (*p* = 0.20, *I*^2^ = 21% and *p* = 0.08, *I*^2^ = 36%, respectively). Although average reduction in body weight and body fat mass were slightly greater after removal of the Tanaka study, they remained modest (Body weight: −1.10 kg [−1.65, −0.56 kg], *p* < 0.001; Body fat mass: −1.41 kg [−2.04, −0.77 kg], *p* < 0.001).

Excluding both comparisons conducted only in males (Tanaka, 2014 [41] and Bowen, 2005 [43]), reduced heterogeneity further (*p* = 0.32, *I*^2^ = 11% and *p* = 0.26, *I*^2^ = 21%, respectively) (Body weight: −1.16 kg [−1.66, −0.66 kg], *p* < 0.001, *n* = 644; Body fat mass: −1.49 kg [−2.06, −0.92 kg], *p* < 0.001, *n* = 521). The average proportion of women in these trials was 90%. 

Excluding studies with duration >12 months [39,40] did not affect heterogeneity or outcomes. Four out of 18 studies were conducted by the same research group, namely Zemel et al. [46,54,56,58]. Removal of the Zemel studies together with Tanaka, 2014 minimally affected the overall result (Body weight: −0.91 kg [−1.50, −0.33 kg], *p* < 0.01, *I*^2^ = 19%; Body fat mass: −1.07 kg [−1.65, −0.48 kg], *p* < 0.001 *I*^2^ = 12%).

The two studies by Anderson, 2005 and 2007 [23,24] (not included in the quantitative meta-analysis because critical data not available in suitable format) did not show differences in body weight and fat mass with milk-based or casein-based meal replacements compared to soy-based meal replacements. However, intervention and control meal replacements were not matched for key nutrients like calcium, protein and fiber which limit direct comparisons. 

Subgroup analysis showed no significant differences in results from studies that specified the use of low-fat or skimmed dairy products [43,45,46,55,56,57] compared to those that did not specify the product type used (Body weight, *p* = 0.39; Fat mass, *p* = 0.60).

#### 3.4.2. Energy Restriction with Resistance Training

A total of 307 participants (267 in dairy food studies; 40 in dairy supplement studies) in 7 comparisons (6 using dairy food; 1 using dairy supplement) (from 6 studies (5 using dairy food; 1 using dairy supplements)) were included in the analysis for body weight and body fat mass change during energy restriction with resistance training. 

Energy restricted diets which incorporated a resistance training component did not result in any difference in changes in body weight (Figure 2) or body fat mass (Figure 3) between the dairy or control groups (Body weight: 0.36 kg [−0.34, 1.07 kg], *p* = 0.31, low heterogeneity *p* = 0.20, *I*^2^ = 30%; Body fat mass: 0.09 kg [−1.20, 1.37 kg], *p* = 0.90, substantial heterogeneity *p* < 0.01, *I*^2^ = 82%). Only one study used supplements thus precluding any formal comparison between whole dairy food and supplements. The Longland, 2016 study [42] was different from other studies with respect to several characteristics; it was a male-only study (other studies 95% female), it used dairy supplements, and it was the shortest duration (4 weeks vs. 12–16 weeks). Removing Longland, 2016 did not substantially alter heterogeneity or overall results. Similarly, removing Thomas, 2011 [44] that used a small dairy serve difference did not change heterogeneity or overall results. 

### 3.5. Effects of Dairy Intake on Body Lean Mass during Energy Restriction

**Figure 4:**
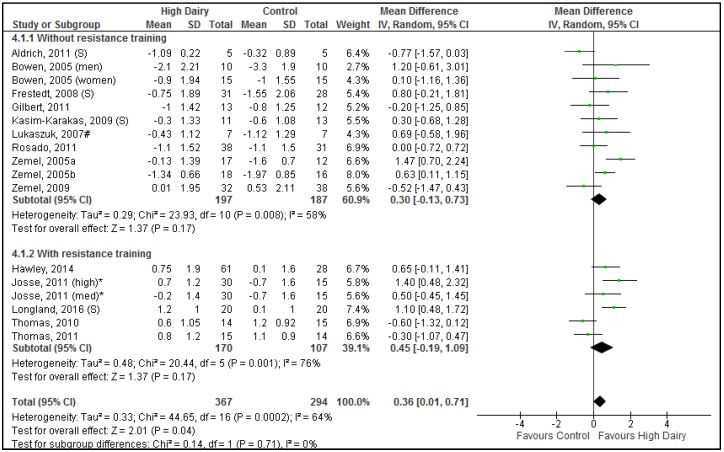
Forrest plot of mean (95% CI) weighted differences in body lean mass (kg) between high dairy and control groups stratified for sub-groups with resistance training vs. without resistance training. (S) The intervention was dairy supplements; # The comparison group was soy protein; * Sample size adapted for multiple comparisons to avoid data duplication; med, medium dairy group; high, high dairy group.

A total of 661 participants (528 in dairy food studies; 133 in dairy supplement studies) in 17 comparisons (13 using dairy food; 4 using dairy supplement) (from 15 studies (11 using dairy food; 4 using dairy supplements)) were included in the analysis for lean body mass change during energy restriction. Resistance training sub-groups did not differ significantly (Chi^2^ = 0.14, *p* = 0.71) (Figure 4) hence the results described are from the resistance training and non-resistance training studies combined. Overall (when all studies are pooled), reductions in lean mass associated with weight loss were significantly less with increased dairy intake compared to control interventions (0.36 kg [0.01, 0.71 kg], *p* = 0.04) (Figure 4). The weighted average reduction in lean mass in the control groups was −0.56 ± 1.54 kg; 95% CI −0.38, −0.74 whereas the weighted average reduction in the dairy groups was −0.12 ± 1.57 kg; 95% CI 0.04, −0.28, hence lean mass loss was attenuated by ~75% in the dairy intervention groups. Substantial heterogeneity was seen for lean mass results (*p* < 0.001, *I*^2^ = 64%). The heterogeneity did not appear to be associated by any one study or specific study characteristic. Dairy food vs. dairy supplement studies did not differ significantly (*p* = 0.98). 

### 3.6. Effects of Dairy Dose on Body Composition Outcomes

Meta-regression was conducted to examine the study-level relationship between the differential dairy food intake (difference between dairy food intake in the intervention group compared to the control group) and mean body weight outcome for those 15 studies conducted without resistance training, and using a dairy food intervention. There was no significant univariate relationship between differential dairy food intake and body weight outcome (regression coeff −0.28, 95% CI −1.48, 0.93) (Figure S1). This result was qualitatively unchanged when other co-variates (mean age, mean baseline BMI, study duration, % of female participants) were included in the model (regression coeff 0.24, 95% CI −1.05, 1.53). Thirteen studies contributed data to the full regression model because mean baseline BMI was not available for Zemel et al. [58] and Van Loan et al. [52]. None of the included variables in the full model were significantly associated with body weight outcome except for % of female participants (regression coefficient −0.04, 95% CI −0.07, −0.01). 

In relation to body fat mass, 13 studies were available which measured difference in change in fat mass by dairy food intake without resistance training. There was no significant univariate relationship between the dairy food intake contrast (dose difference) and body fat mass outcome in these studies (regression coefficient −0.16, 95% CI −1.63, 1.31). After adjustment for other covariates (mean age, mean baseline BMI, study duration, % of female participants; *n* = 11 studies) this result did not change (regression co-efficient 0.24, 95% CI −1.76, 2.24).

In examining lean body mass, trials based on differences in whole dairy food intake which included resistance training were combined with studies that did not include resistance training in meta-regression (*n* = 13 studies). In univariate analysis, difference in lean body mass was not significantly associated with dairy food dose difference used in the studies (regression co-efficient 0.24, 95% CI −0.13, 0.60). After adjustment for other covariates (mean age, mean baseline BMI, study duration, % of female participants, whether study included resistance training; *n* = 13 studies), the association remained not significant (regression co-efficient 0.10, 95% CI −0.30, 0.50). 

### 3.7. Publication Bias

Examination of funnel plots indicated moderate asymmetry (lack of smaller trials (trials with larger SEs) reporting a negative results) suggesting presence of a trend for publication bias in body weight, fat mass and lean mass (Figure S2). 

### 3.8. Quality of the Total Body of Evidence

Table 2 summarizes the process of rating the quality of the body of evidence according to the GRADE guidelines and provides a rating that reflects the degree of certainty in the relationship between dairy intake and body weight or composition.

The degree of certainty that increased intake of dairy products under conditions of energy restriction without resistance training enhances body weight and body fat mass loss in 18–50-year-old overweight/obese participants is rated moderate. This is because the studies were conducted primarily in women and there is some evidence that trial results are different for men compared to women. However, when considering the relationship in women (excluding the two male-only trials, resulting in a sample with an average of 90% women) the degree of certainty was rated as high. The degree of certainty in the relationship when resistance training was imposed was rated as low to moderate. This is due to a limited number of studies and substantial inconsistency between studies. Further research may change the effect estimate and rating. The degree of certainty that increased intake of dairy will reduce the amount of lean mass lost under conditions of energy restriction was rated as moderate. The rating was mainly determined by the high degree of inconsistency observed between studies.

## 4. Discussion

The findings of this study were that under hypocaloric conditions, a high dairy product intervention resulted in greater body weight and fat mass loss, and attenuated lean mass loss compared to control interventions. There was no evidence for a difference in outcome with ‘dose’ of dairy food with most trials using a contrast of 2 to 3 servings of dairy food a day. These effects were evident with both dairy foods and dairy supplements. However, under energy restricted conditions with the inclusion of resistance training, dairy consumption had no additional effects on body weight or fat mass loss and results for fat mass were inconsistent between studies. As the majority of participants in individual studies were females further research in males is required to investigate sex effects.

The results are in agreement with recently published meta-analysis showing greater weight and fat mass loss in the context of energy restriction [9,10] and reduced lean mass loss with dairy intake compared to control [10]. The current meta-analysis advances this prior research by including two trials missed in the previous meta-analyses [45,57], including more recently published trials [41,50] and trials using dairy protein supplements [21,42,47,48,49] and targeting the specific age group of 18–50 years. In addition, the current meta-analysis considered the totality of the evidence and rated the degree of certainty in the relationship between dairy and body weight/composition. 

### 4.1. Energy Restriction without Resistance Training on Body Weight and Fat Mass Loss

Results from studies where dairy products were consumed in conjunction with an energy restricted diet without resistance training were relatively consistent in showing greater reductions in body weight and fat mass following dairy intake. The effects were evident with both dairy food and dairy supplements. The greater body weight loss (~1 kg) over a median period of 16 weeks was primarily due to greater fat mass loss. Whilst modest, this greater level of weight loss is clinically relevant. A reduction in body weight of 1 kg was associated with a 16% risk reduction of developing diabetes in 25–84-year-old overweight/obese participants [62]. 

One study by Tanaka et al. [41] resulted in high levels of heterogeneity. Removal on this study reduced heterogeneity from substantial to low levels. Tanaka et al. [41] was different with respect to several characteristics compared to other studies: it included only men whereas most other studies included mostly women (mean 80% women); the difference in dairy serves between intervention and control groups was small (~1.3 serves/day vs. 2+ serves/day in other studies); body composition was assessed using BIA, a method considered less sensitive to assess small changes in body fat [60]; only three quarters of the sample was overweight/obese vs. 100% overweight/obese in other studies and it was the only study conducted in a Japanese population. Variations in some study characteristics may have affected responses to interventions, albeit only mildly since heterogeneity and overall results were not affected by these characteristics during sensitivity analysis. Responses in fat mass loss were smaller and more variable in the two long-term studies (≥12 month) [39,40] and in studies where participants may have had high dairy or calcium intakes at baseline [20,39,43]. A recent meta-analysis suggested a calcium threshold of ~600–700 mg/day above which no further benefit of additional intake on body composition is observed [63]. The pilot study (*n* = 7) by Lukaszuk et al. used soy-milk with added leucine as control, resulting in matched intakes of protein and calcium between dairy and control groups and failed to show additional benefit of dairy consumption on body weight or fat mass loss [45].

Studies were mostly conducted in women (women comprised 80% of the total sample) and comparisons examining men only [41,43] tended to favor the control treatment. However, the male sub-group in Bowen, 2005 [43] was very small (*n* = 10/group) and Tanaka, 2014 [41] differed with regard to several other characteristics which limits the conclusions that can be drawn regarding the effects of dairy on body weight and composition in men. Sex differences in body composition changes in response to weight loss have been suggested [64], but it is unclear why sex may modulate changes in body weight and composition in response to dairy consumption. Further research is needed to investigate the effect of dairy consumption on body weight and composition changes in men. 

The protein component of dairy is a candidate for an important role in its effects on body weight and fat mass loss, possibly related to the greater thermic effects of protein [65,66]. This is supported by the three dairy (or whey) protein supplement studies that showed benefits for body weight and fat mass loss [47,48,49]. All three studies reported higher dietary protein intake compared to the control groups (1.07 vs. 0.61 g/kg [49]; 1.46 vs. 0.78 g/kg [48] and 0.81 vs. 0.61 g/kg [47]). Moreover, findings from Frestedt et al. indicate that even small increases in protein intake of just 0.2 g/kg using whey fraction high in leucine can have beneficial effects for groups who may not otherwise be meeting their RDA for protein [47]. A pilot study where dairy milk was compared to soy milk (protein and calcium content matched between interventions) did not have any additional weight loss effects [45]. The meta-analysis by Wycherley et al. [8] further supports a role for protein by showing modest benefits of high-protein diets from different sources in conjunction with energy-restricted diets for reducing body weight (−0.79 kg [−1.50, −0.08 kg]) and fat mass (−0.87 kg [−1.26, −0.48 kg]). Further accumulating evidence suggests that dairy protein, particularly whey protein, may affect body weight through regulation of food intake and appetite [4]. Indeed, Gilbert showed that milk supplementation induced a smaller increase in desire to eat and hunger, suggesting attenuation of weight loss-related increase in appetite [20]. However, in the context of prescribed energy restriction used in the studies examined, where food and energy intake is strictly controlled therefore not allowing for *ad libitum* feeding, the appetite regulating mechanism may be less operative. From the current evidence it is not possible to ascertain whether the effects of dairy interventions were directly attributable to dairy protein or to increased intake of total dietary protein.

It is also possible the high calcium content of dairy may have contributed to the weight and fat mass reducing effects. Dairy food intervention groups, where the differences in dairy serves were >2 servings/day, consumed ~582–1862 mg/day more calcium compared to control groups while protein intake in most of these studies were held constant. Using a mixed-model regression analysis including 18 observational studies that examined dietary calcium (with the majority of dietary calcium derived from dairy products), Dougkas et al. [4] showed that an increased calcium intake from 400 to 1200 mg/day was associated with a reduction in body mass index (BMI) from 25.6 to 24.7 kg/m^2^. The most cited mechanism for the effect of calcium on weight loss, demonstrated in cell-culture and animal studies, involves the influence that ingested calcium has on intracellular calcium and subsequently adipocyte lipid metabolism by reducing de novo lipogenesis and increasing fat oxidation. However, studies in humans have failed to support this hypothesis [4]. Alternatively, a more plausible mechanism may be that high dairy calcium increases faecal fat excretion. A meta-analysis by Chirstensen et al. [2] indicated that dairy calcium consumption of 1241 mg increased faecal fat excretion by 5.2 g/day (CI 1.6, 8.8 g/day) compared with low dairy calcium intake (<700 mg/day). The authors estimated that this level of fat excretion would translate into 1.9 kg body fat or 2.2 kg body weight loss over a year. As noted by Dougkas et al. [4], other investigators have suggested that the impact of calcium on fat absorption may be protein dependent suggesting that the impact of calcium from dairy may be greater than supplemental calcium. 

Furthermore, dairy is a rich source of MCT, C8:0 (caprylic acid) and C10:0 (capric acid), which have been shown to increase energy expenditure, lipid oxidation and satiety [67]. A recent meta-analysis showed dosages ranging from 2 to 54 g/day produced small reductions in body weight (−0.51 kg [−0.80, −0.23 kg]) compared to long-chain triacylglycerols over study periods of 1–4 months [68]. Dairy is also a source of the fatty acid CLA, which has been shown to affect body weight and fat mass. The meta-analysis by Onakpoya et al. [69] showed small reductions in body weight (−0.70 kg [−1.09, −0.32 kg]) and fat mass (−1.33 kg [−1.79, −0.86 kg]) with dosages ranging from 2.4 to 6 g/day over an average period of 8 months. MCT and CLA may therefore contribute to weight reducing effects, but it is unlikely that the small amounts of these fatty acids in dairy will have measurable weight reducing effects. Three servings of dairy/day would supply ~1 g C8:0 + C10:0 (RMIT fatty acid database, FoodWorks, 2009, Xyris Software, Kenmore Hills, Queensland, Australia) and ~0.1 g CLA [70] while the amounts will be negligible in low-fat/skimmed dairy products. Several other dairy constituents such as lactose, protein and their peptide derivatives may also affect body weight through the regulation of food intake and appetite although there is a paucity of evidence from well-designed intervention studies that closely controlled energy intake to confirm the mechanisms [4].

### 4.2. Energy Restriction with Resistance Training on Body Weight and Fat Mass Loss

In contrast to the studies examining energy restricted diets without resistance training, the small number of comparisons (*n* = 7) that included a resistance training component to the energy restricted diets did not reveal any beneficial effects of dairy on body weight or fat mass. Substantial heterogeneity was seen between studies for fat mass explained by inconsistent findings, with some studies favoring dairy intake [12,42], some favoring control treatment [22,53] and others showing no effect [12,44,50]. It is possible the potent effects of combined energy restriction and resistance training may have maximized the weight and fat loss effects that precluded any further modest influence of dairy from being observed. Greater intakes of dairy, providing greater amounts of protein and calcium, may be required in conjunction with resistance training to have a measurable effect on body fat mass. Comparisons that showed greater body fat mass loss provided high dosages of dairy resulting in considerably greater intakes of protein [12,42] and calcium [12] compared to the control groups. Josse et al. [12] showed a dose response effect with significant greater fat mass loss in the high dairy compared to the low dairy groups (6 servings/day difference) whereas the difference was not significant between the medium and low dairy groups (3 servings/day difference). Protein and calcium intake in the high compared to the low dairy groups were significantly greater (protein: 28 vs. 16% E, 1.3 vs. 0.72 g/kg; calcium: 1840 vs. 299 mg/day) [12] whereas protein intake was comparable between moderate and low dairy groups (18 vs. 16% E; 0.84 vs. 0.72 g/kg). Longland et al. [42] provided whey protein isolate to achieve protein intakes of 2.4 g/kg/day vs. 1.2 g/kg/day in the control group. Furthermore, high baseline intakes of calcium (>700 mg/day) in some studies [50,53] may have counteracted any additional body weight or fat mass loss [63]. 

The degree of certainty in the relationship between dairy intake and body weight/fat mass when resistance training was imposed was rated as low to moderate due to limited number of studies and substantial heterogeneity between studies. Hence, before any conclusion can be made further research is required to determine the role of dairy on body weight/fat mass in conjunction with energy restriction and resistance training. 

### 4.3. Attenuation of Loss in Lean Mass

Incorporation of dairy within an energy restricted diet attenuated the loss of lean mass compared to control interventions. Whole dairy and particularly the whey protein component of dairy has a high leucine content which is a key essential amino acid for triggering muscle protein synthesis [71]. The reduction in loss of lean mass was modest, 0.36 kg [0.01, 0.71 kg], which likely explains why most individual studies could not demonstrate a significant effect while the pooled result of the meta-analysis showed significant effects likely due to enhanced statistical power. Despite the relatively modest effect size, lean mass loss was reduced by about ~75% which may have clinical relevance for regulating resting energy expenditure, protein metabolism and postprandial glucose uptake [8]. 

These results were observed despite most studies prescribing standard amounts of protein (within recommended intake ranges) and similar protein intakes between dairy and control groups. However, the greatest effects of dairy on lean mass were observed in studies where protein intake was higher in the dairy groups (>~1.2 g/kg/day) compared to control groups (<~1.2 g/kg/day) and energy restriction was combined with resistance training [12,42,50]. Although, the independent effects of dairy protein on lean mass cannot be ascertained from this systematic literature review, whey protein has been shown to be more effective at preserving muscle protein synthesis compared to soy protein [72]. Whey protein supplementation attenuated the decline in postprandial rates of muscle protein synthesis after weight loss compared to soy protein or a carbohydrate control [72]. Furthermore, acute mechanistic studies have demonstrated that following resistance exercise training, whey protein induced a superior muscle protein synthesis response compared to casein, soy or carbohydrate [3,73]. Further studies incorporating a control group consuming a different type of protein, e.g., soy protein is needed to determine whether the effects on lean mass are attributable specifically to dairy protein or dietary protein intake in general. Due to the high degree of heterogeneity observed between studies the overall quality of the evidence for the relationship between dairy intake and lean mass under conditions of energy restriction was rated as moderate. 

### 4.4. Dose

Across studies conducted under hypocaloric conditions dose of dairy food did not show a linear relationship with mean body weight or composition change differences between dairy and control groups. Improvements in body weight and composition were seen with dairy food differences ranging from 2 to 4 servings/day. However, meta-regression of RCTs is prone to confounding due to differences between trials, the range of dose differences was small, background intake of dairy food was often not stated, nor compliance with required intake. Ideally dose-response would be investigated in specifically designed trials. Josse et al. [12] showed greater fat mass reductions and lean mass increases with 6–7 servings of dairy food (milk, cheese, yoghurt)/day compared to 3 servings/day and 0–1 servings/day under conditions of energy restriction with resistance training for 16 weeks. 

The absence of a detectable dose-response effect may detract from inferring a causal relationship between dairy consumption and body weight and composition changes. On the other hand, the lack of a dose-response effect may suggest a threshold amount of dairy that is required to promote favorable body composition changes above which no further benefit is attained. Onakpoya et al. [63], from their meta-analysis, suggested a calcium threshold of ~600–700 mg/day (~2 servings of dairy) above which no further benefit of additional intake of calcium on body composition is observed.

### 4.5. Strengths and Limitations

Strengths of this systematic literature review include strict adherence to the Cochrane systematic review guidelines; acquisition of a sizeable and comprehensive study data-set for meta-analysis through contacting authors or estimating mean changes and SDs for missing data (only data from 2 out of 27 studies were not in a suitable form for inclusion in the meta-analysis); conducting dose-response analysis; considering the totality of the evidence by rating the degree of certainty in the relationship between dairy and body weight/composition using GRADE guidelines; and conducting a priori sub-group analyses and extensive sensitivity analyses to identify potential causes of inconsistencies between studies. Most studies included were of high quality and inclusion of studies that examined both dairy foods and supplements provides a comprehensive understanding of the effects of dairy intake on body weight and composition. Restriction of the review to younger adults may have improved homogeneity, but limits generalizability for older individuals. Other limitations were the finding that studies were largely of women, hence the results cannot be readily generalized to men; the effects of different dairy sources could not be distinguished, e.g., H milk vs. cheese vs. yoghurt because different dairy sources were typically not compared within studies; some a priori sub-group analysis could not be performed (i.e., effects of study duration and sex differences) due to a limited number of studies with differentiating characteristics; the impact of habitual dietary intakes could often not be assessed because they were not reported; and few studies reported compliance to the desired interventions. Furthermore, a high degree of heterogeneity was seen between studies for the relationship between dairy intake and lean mass which could not be explained; and a trend for publication bias was identified. 

## 5. Conclusions

The evidence shows modest benefits for dairy product consumption (≥2 servings of dairy food/day or on average 55 g whey protein/day) on body weight and composition (fat mass loss and lean mass loss attenuation) over a median period of 16 weeks in the context of energy restricted diets in overweight and obese adults aged 18–50 years. The degree of certainty in the relationship between dairy intake and body weight and fat mass under conditions of energy restriction in 18–50-year-old women was rated as high. Due to considerable heterogeneity the relationship between dairy intake and lean mass was rated as moderate. In studies that imposed resistance training the degree of certainty rating for body weight and body fat mass outcomes were low to moderate due to substantial heterogeneity and limited number of studies; hence requiring further investigation. Since the majority of participants examined were females, further research in males is required to investigate sex effects.

## Figures and Tables

**Table 1:** Characteristics of randomized controlled trials included in the systematic literature review ^1^.

Reference ^2^ (Country)	Quality Score ^3^	Population				Intervention							
		*N* ^4^	Age (Year)	Description (BMI) ^5^	Female (%)	Dairy	Control	Estimated Dose Difference ^6,7^	Calorie Restriction	Iso-Caloric ^8^	Exercise ^9^	Duration (Weeks)	Outcomes Assessed
**Dairy Food Studies**													
Anderson, 2005 [24] (USA)	11	90 (52)	18–65	Overweight/obese BMI = 27–40	88	2× milk-based meal replacement (Slim-Fast^®^)/day	5× soy-based meal replacements (Scan-Diet™)/day	2 servings	Diets = 5020 kJ	Yes	No	12	BW ^10^
Bowen, 2005 [43] (Australia)	8	(50)	20–65	Overweight/obese BMI = 27–40	60	4.5 serves dairy/day (skim milk, reduced fat cheese, yoghurt, skim milk powder)	0.5 serves dairy/day	4 servings	Caloric restriction (12 weeks): Diet = 5500–7000 kJ Eucaloric (4 weeks)	Yes	No	16	BW, FM, LM
Faghih, 2011 [55] (Iran)	7	50 (42)	20–50	Healthy pre-menopausal overweight/obese women BMI = 25–40	100	3× 220 mL low-fat milk/day	Control diet providing 500 mg/day dietary Ca	2.64 servings	−2092 kJ	Yes	No	8	BW, FM
Gilbert, 2011 [20] (USA)	10	41 (25)	25–50	Healthy overweight/obese low Ca consumers (<800 mg/day) BMI = 27–42	100	1 serving (568 mL providing 1000 mg Ca) milk supplement/day (Lactancia Addition Nature 35% plus de Ca (1% fat); Parmalat Canada, Toronto, ON, Canada)	1 serving (463 mL) Placebo (Rice Dream; Hain Celestial Canada, Toronto, ON, Canada)	2.3 servings	−2508 kJ	Yes	No	24	BW, FM, FFM
Harvey-Berino, 2005 [39] (USA)	9	55 (44)	18–60	Overweight/obese adults with low dairy (<1 serve/day) and Ca (<500 mg/day) intake. Baseline dairy intake ~1.4 servings/day BMI = 25–34.9	91	3–4 (3.2 ± 1.1) servings dairy/day (milk, yogurt, cheese) providing 1200–1400 mg Ca/day	1 (0.85 ± 0.4) serving dairy/day providing 400–500 mg Ca/day.	2.7 servings	−2092 kJ	Yes	No	52	BW, FM
Hawley, 2014 [50] (Australia)	9	111 (89)	35–59	Health overweight/obese sedentary low dairy consumers (1.3 serves/day) BMI = 27–40	71	4 + (3.8 ± 0.7) serves dairy/day	1–2 (1.0 ± 0.3) serves dairy/day	2.8 servings	−1046 kJ	Yes	RT: 3×/week AT: 4×/week	16	BW, FM, LM
Josse, 2011 [12] (Canada)	13	90 (78)	19-45	Pre-menopausal overweight/obese low dairy consumers BMI = 27–40	100	High dairy: 6–7 servings dairy/day (milk, cheese, yoghurt)	Low diary: 0–1 servings dairy/day	6 servings	−2092 kJ	Yes	RT: 2×/week + AT: 7×/week	16	BW, FM, LM
						Medium dairy: 3–4 servings dairy/day (milk, cheese, yoghurt)		3 servings					
Lukaszuk, 2007 [45] (USA)	10	18 (14)	18–45	Healthy pre-menopausal overweight/obese low Ca consumers (<600 mg/day) BMI = ~36	100	3 cups/day (720 mL) skim milk	3 cups/day (720 mL) light soy milk + added soy protein to equate protein of milk	3 servings	−2092 kJ	Yes	No	8	BW, FM, FFM
Rosado, 2011 [51] (Mexico)	11	93 (69)	25–45	Obese low dairy consumers (<3 servings/day) BMI ≥ 30	100	3 servings milk/day. No other dairy.	0 servings of dairy/day.	3 servings	−2092 kJ	Yes	No	16	BW, FM ^10^, LM
Summerbell, 1998 [38] (UK)	13	28 (20)	>17	Healthy overweight/obese BMI ≥ 27	79	Milk (full-cream/skim) to provide 3.4 MJ (nil other food)	Conventional balanced diet providing 3.4 MJ/day	7.3 servings	Diet = 3.4 MJ	Yes	No	16	BW
Tanaka, 2014 [41] (Japan)	12	213 (200)	20–60	≥2 components of metabolic syndrome BMI = average ~27	0	400 g dairy/day (milk, yoghurt)	Low dairy: <0.5 servings dairy/day	1.3 servings	−1255 kJ	Yes	No	24	BW, FM
Thomas, 2010 [22] (USA)	12	35 (29)	29–45	Overweight/obese low dairy consumers (≤1 serving/day) BMI = 25–30	100	≥3 servings dairy/day providing 1200 mg Ca/day	≤1 serving dairy/day providing 500 mg Ca/day	3 servings	−1046 kJ	Yes	RT: 3×/week	16	BW, FM, FFM
Thomas, 2011 [44] (USA)	10	35 (29)	29–45	Overweight, non-RT BMI = 25–30	100	2× 170 g fat free yoghurt 3×/week—20 min before exercise and immediately after exercise	2× sucrose beverage 3×/week—20 min before exercise and immediately after exercise	0.75 servings	−1046 kJ	Yes	RT: 3×/week	16	BW, FM, LM
Thompson, 2005 [40] (USA)	13	59 (48) (adherers: 36)	25–70	Obese adults BMI = 30–40	86	4 servings (3.13 achieved) dairy/day (2 as fluid milk)	2 servings (1.38 achieved) dairy/day	1.8 servings	−2092 kJ	Yes	AT: 30 min 4×/week	48	BW, FM
Torres, 2010 [57] (Brazil)	11	50 (39)	22–55	Obese low Ca consumers (<500 mg/day) of multi-ethnic origin BMI = 30–34.9	90	60 g/day (2 servings) of non-fat powdered milk (1200–1300 mg Ca/day)	Low-Ca diet (<500 mg Ca/day)	2 servings	−3347 kJ	Yes	No	16	BW
Van Loan, 2011 [52] (USA)	7	78 (71)	19–50	Overweight/obese low dairy consumers (≤1 serving dairy/day) BMI = 28–37	77	3–4 servings dairy/day (milk, yogurt, cheese) providing 1339 mg Ca/day	≤1 serving dairy/day providing 460 mg Ca/day	3 servings	−2092 kJ	Yes	No	15	BW, FM, LM ^10^
Wagner, 2007 [53] (USA)	9	~42 (30)	19–53	Pre-menopausal overweight BMI = 26–40	100	Low-fat milk providing 800 mg calcium/day	Placebo capsules (cellulose)	2.7 servings	−2092 kJ	Yes	RT + AT: 3×/week	12	BW, FM
Zemel, 2004 [58] (USA)	8	28 (21)	18–60	Healthy overweight/obese BMI = 30–39.9	81	3 serves of dairy providing 1200–1300 mg Ca/day + placebo	<1 serve of dairy providing 400–500 mg of Ca/day + placebo (content NR)	2.5 servings	−2092 kJ	Yes	No	24	BW, FM
Zemel, 2005a [56] (USA)	9	36 (29)	26–55	Healthy obese, low dairy consumers BMI = 30–40	86	3 serves low-fat dairy/day providing 1200 mg/day Ca, one as fluid milk.	Habitual diet 0–1 serves diary/day providing 500 mg/day Ca.	~2.5 servings	−2092 kJ	Yes	No	24	BW, FM, LM
Zemel, 2005b [46] (USA)	9	38 (34)	18–50	Healthy obese BMI = 30–39.9	79	3 serves (3× 170 g) fat free yoghurt (Yoplait Light)	0–1 serves dairy/day + 3 serves of sugar-free, Ca-free, gelatin dessert (42 kJ/serve)	~2.0 servings	−2092 kJ	Yes	No	12	BW, FM, LM
Zemel, 2009 [54] (USA)	9	70 (64)	18–35	Healthy overweight/mildly obese, low Ca intake (<600 mg/day) BMI = 25–34.9	77	3 serves dairy/day (full/low-fat milk, cheese, yogurt) providing 1400 mg Ca/day	0–1 serve dairy/day providing 500 mg Ca/day	3.0 servings	−2092 kJ	Yes	No	12	BW, FM, LM
**Dairy Supplement Studies**													
Aldrich, 2011 [48] (USA)	8	12 (10)	40–60	Overweight/obese BMI = 27–32	80	3× 28 g/day serves of Designer Whey (whey protein isolate) + 1.68 serves of milk/day	Assigned control diet including 1.2 serves of dairy/day + Ca tablets to balance Ca intake	84 g	Tailored to promote 0.75 kg weight loss/week. 8 weeks feeding followed by 12 weeks a*d libitum*	Yes, for 8 weeks weight loss phase	No	8	BW, FM, LM
Anderson, 2007 [23] (USA)	10	43 (35)	20–65	Healthy obese BMI = 30–40	100	3× Casein shakes/day (67.5 g protein) (Revival Soy, Physicians Pharmaceuticals Kernersville, NC)	3× Soy shakes/day (61.8 g protein)	67.5	Diets = 4200–5000 kJ	Yes	Physical activity levels of 8400 kJ/week	16	BW ^10^, FM ^10^, LM ^10^
Frestedt, 2008 [47] (USA)	8	106 (59)	25–50	Obese BMI = 30–42	NR	2×/day whey fraction high in leucine (Prolibra ™) 20 min before breakfast and dinner <1 serving of dairy/day	2×/day maltodextrin <1 serving of dairy/day	20 g	−2092 kJ	Yes	No	12	BW, FM, LM
Kasim-Karakas, 2009 [49] (USA)	11	33 (24)	18–45	PCOS BMI = 25–40	100	Whey protein isolate (96% pure) (Glanbia Foods, Twin Falls, ID)	Glucose + maltose + tricalcium phosphate	60 g	−1883 kJ	Yes	No	8	BW, FM, LM
Longland, 2016 [42] (Canada)	12	40 (40)	18–30	Overweight BMI > 25	0	Whey protein isolate (Agropur IsoChill 9010) added to 680 ± 120 mL/day skimmed milk. Total whey consumed: 85 ± 20 g/day. 3–4 beverage/day; one consumed immediately after training.	Maltodextrin added to 530 ± 116 mL/day whole milk. Total whey consumed: 12 ± 9 g/day. 3–4 beverage/day; one consumed immediately after training.	~73 g	40% lower energy than estimated require-ments	Yes	RT + AT: 6×/week	4	BW, FM, LM
Piccolo, 2015 [21] (USA)	9	NR (29)	18–56	Obese with metabolic syndrome BMI = 27–42	100	2×/day 10 g whey-based supplement (Glanbia, Inc.)	2×/day 10 g gelatin-based protein supplement (Glanbia, Inc.)	20 g	Tailored to achieve 5%–10% reduction in body weight (−2740 ± 584 kJ)	Yes	No	8	BW

AT, aerobic exercise training; Ave, average; BMI, body mass index; BW, body weight; Ca, Calcium; CHO, carbohydrate; FFM, fat free mass; FM, fat mass; LM, lean mass; NR, not reported; PCOS, polycystic ovary syndrome; RT, resistance exercise training; ^1^ All included studies had a randomized controlled parallel study design; ^2^ First author, year of publication; ^3^ Rated using the Health Canada Quality appraisal tool for intervention studies [13]. See details of quality rating in Table S3; ^4^ Enrolled (completed); ^5^ Range or ~mean of baseline BMI (kg/m^2^); ^6^ Difference in servings of daily dairy intake between dairy intervention and control intervention. Serving estimations were based on Australian standard serves (250 mL milk, 200 g yoghurt, 40 g hard, firm, soft and low fat cheese, 120 g cottage and ricotta cheese, 200 g custard, 30 g powdered milk). Where only calcium provided by dairy was reported, serves of dairy were estimated based on 300 mg calcium/dairy serve [61]; ^7^ Difference in grams of daily dairy supplement intake between dairy intervention and control intervention; ^8^ The two diets/supplements were similar in energy content; ^9^ An exercise program was prescribed, including either resistant or aerobic training or both as part of the interventions (the same program in both dairy and control groups); ^10^ Data not in suitable format for meta-analysis.

**Table 2:** Rating of the quality of the body of evidence using GRADE guidelines.

Quality Assessment of Body of Evidence							Number of Participants		Effect Estimate	Quality (Degree of Certainty)
Number Studies ^1^	Design	Risk of Bias	Inconsistency	Indirectness	Imprecision	Publication Bias	Dairy	Control	Mean Difference (95% CI) (kg)	
1. Increased intake of dairy products under conditions of energy-restriction without resistance training enhances body weight loss in 18–50-year-old overweight/obese participants.										
18 (19)	RCT	Moderate ^2^	Low ^3^	Moderate ^4^	Low	Moderate	443	421	−0.92 [−1.63, −0.20]	⊕⊕⊕ Moderate ^5^
2. Increased intake of dairy products under conditions of energy-restriction without resistance training enhances body weight loss in 18–50-year-old overweight/obese women.										
17 (17) ^6^	RCT	Moderate ^2^	Low ^7^	Low	Low	Moderate	331	313	−1.16 [−1.66, −0.66]	⊕⊕⊕⊕ High
3. Increased intake of dairy products under conditions of energy-restriction without resistance training enhances body fat mass loss in 18–50-year-old overweight/obese participants.										
15 (16)	RCT	Moderate ^2^	Low ^8^	Moderate ^4^	Low	Moderate	374	367	−1.24 [−2.10, −0.37]	⊕⊕⊕ Moderate ^5^
4. Increased intake of dairy products under conditions of energy-restriction without resistance training enhances body fat mass loss in 18–50-year-old overweight/obese women.										
14 (14) ^6^	RCT	Moderate ^2^	Low ^9^	Low	Low	Moderate	262	259	−1.49 [−2.06, −0.92]	⊕⊕⊕⊕ High
5. Increased intake of dairy products under conditions of energy-restriction in conjunction with resistance training enhances body weight loss in 18–50-year-old overweight/obese participants.										
6 (7)	RCT	Moderate ^10^	Low ^11^	Moderate ^4^	Moderate ^12^	Moderate	187	120	0.36 [−0.34, 1.07]	⊕⊕⊕ Moderate
6. Increased intake of dairy products under conditions of energy-restriction in conjunction with resistance training enhances body fat mass loss in 18–50-year-old overweight/obese participants.										
6 (7)	RCT	Moderate ^10^	Substantial ^13^	Moderate ^4^	High ^12^	Moderate	187	120	0.09 [−1.20, 1.37]	⊕⊕ Low ^14^
7. Increased intake of dairy products under conditions of energy-restriction reduces the loss in lean mass associated with energy restriction in 18–50-year-old overweight/obese participants.										
15 (17)	RCT	Moderate ^15^	Substantial ^16^	Moderate ^4^	Low	Moderate	367	294	0.36 [0.01, 0.71]	⊕⊕⊕ Moderate ^17^

GRADE, Grading of Recommendations Assessment, Development and Evaluation; ^1^ Number of studies (total number of comparisons from studies); ^2^ Quality scores were high (≥8) with an average score of 9 out of 15 and only 2 studies scoring <8 (both scored 7). Sensitivity analysis involving exclusion of trials with high risk of bias showed no impact on outcomes. Due to the nature of the interventions (whole dairy food in 14/18 studies) blinding was difficult to achieve. Most studies (78%) did not report whether allocation was concealed. Attrition bias was reported or unclear in 8 studies; ^3^ Removal of one study (Tanaka, 2014) reduced heterogeneity from substantial to low levels (*p* = 0.20, *I*^2^ = 21%); Point estimates for most studies favored dairy; CI overlapped for the majority of studies; ^4^ All studies were conducted in the population of interest (overweight/obese 18–50 years). However, studies were conducted mainly in women and cannot readily be generalized to men; ^5^ This grade supports studies mostly conducted in women. Further research focusing on men may have an impact on the grade; ^6^ Removed male-only comparisons (Tanaka, 2014 [41], Bowen, 2005 (men) [43]). The average proportion of women in the remaining studies was 90%; ^7^ Low heterogeneity (*I*^2^ = 11%); ^8^ Removal of one study (Tanaka, 2014) reduced heterogeneity from substantial to low levels (*p* = 0.09, *I*^2^ = 36%); Point estimates for most studies favored dairy; CI overlapped for the majority of studies; ^9^ Low heterogeneity (*I*^2^ = 21%); ^10^ Quality scores were high (≥8) for all studies with an average score of 11 out of 15. Due to the nature of the interventions (dairy food) blinding was difficult to achieve; ^11^ Low heterogeneity (*I*^2^ = 30%); ^12^ 95% CI cross zero; CI for body fat mass wide.; ^13^ Substantial heterogeneity (*I*^2^ = 82%); ^14^ Limited number of studies conducted and results inconsistent; further research may have an impact on the grade; ^15^ Quality scores were high (≥8) for all studies with an average score of 10 out of 15. Due to the nature of the interventions (dairy food) blinding was difficult to achieve; ^16^ Substantial heterogeneity (*I*^2^ = 64%); ^17^ Certainty is particularly affected by inconsistency between studies; most studies have been conducted in women although a small study in men-only was recently published with results in a similar direction than this meta-analysis [42]. Hence, further research may have an impact on the grade.

## Supplementary Materials

The following are available online at http://www.mdpi.com/2072-6643/8/7/394/s1, Table S1: Baseline protein and calcium intakes stratified by intervention group as reported by study participants; Table S2: Daily nutrient intakes from intervention diets as reported by study participants; Table S3: Quality appraisal of included studies; Figure S1: Mean body weight difference (kg) between high dairy and control groups by difference in daily serves of dairy food intake for trials using whole dairy foods (*n* = 15). The size of the circle is proportional to the inverse of the within-study variance of effect size*.*; Figure S2: Funnel plot for A. Body weight (kg); B. Body fat mass (kg); C. Body lean mass (kg); SE, standard error; MD, mean weighted difference between high dairy and control groups.

## Abbreviations

The following abbreviations are used in this manuscript:

ADP	Air displacement plethysmography
AT	Aerobic training
BIA	Bioelectrical impedance analysis
BMI	Body mass index
Ca	Calcium
CHO	Carbohydrate
CI	Confidence interval
CLA	Conjugated linoleic acid
CT	Computed tomography
DEXA	Dual energy X-ray absorptiometry
% E	Percentage of total energy intake
ICTRP	International Clinical Trials Registry Platform Portal
ITT	Intention-to-treat
MCT	Medium-chain triacylglycerols
MeSH	Medical subject heading
MR	Meal replacement
MRI	Magnetic resonance imaging
NR	Not reported
PCOS	Polycystic ovary syndrome
RCT	Randomized controlled trial
RT	Resistance training
