# Supplementary material for: Dairy Intake Enhances Body Weight and Composition Changes during Energy Restriction in 18–50-Year-Old Adults—A Meta-Analysis of Randomized Controlled Trials
Source: Nutrients. 2016 Jul 1;8(7):394. doi: 10.3390/nu8070394 (PMC4963870; doi:10.3390/nu8070394)
Supplement: Supplementary file 1 [file nutrients-08-00394-s001.docx]

Supplementary Materials: Dairy Intake Enhances Body Weight and Composition Changes during Energy Restriction in 18–50-Year-Old Adults—A Meta-Analysis of Randomized Controlled Trials

Welma Stonehouse, Thomas Wycherley, Natalie Luscombe-Marsh, Pennie Taylor,
Grant Brinkworth and Malcolm Riley

**Table S1.** Baseline protein and calcium intakes stratified by intervention group as reported by study participants.

| **Reference ^1^** | **Protein (% E)** | | **Protein (g/kg)** | | **Calcium (mg)** | |
| --- | --- | --- | --- | --- | --- | --- |
|  | **Dairy** | **Control** | **Dairy** | **Control** | **Dairy** | **Control** |
| **Dairy Food** |  |  |  |  |  |  |
| Anderson, 2005 [[1](#_ENREF_24)] | NR | NR | NR | NR | NR | NR |
| Bowen, 2005 [[2](#_ENREF_43)] (men) | NR | NR | NR | NR | 899 | 935 |
| Bowen, 2005 [[2](#_ENREF_43)] (women) | NR | NR | NR | NR | 787 | 737 |
| Faghih, 2011 [3] | NR | NR | NR | NR | 485 | 513 |
| Gilbert, 2011 [[4](#_ENREF_20)] | 18.6 | 17.4 | 1.08 ^3^ | 0.94 ^3^ | 687 | 660 |
| Harvey-Berino, 2005 [[5](#_ENREF_39)] (USA) | NR | NR | NR | NR | 736 | 744 |
| Hawley, 2014 [[6](#_ENREF_50)] (High dairy + medium CHO) | 20 | 21 | 1.0 | 1.2 | 793 | 795 |
| Hawley, 2014 [[6](#_ENREF_50)] (High dairy + high CHO) | 22 |  | 1.1 |  | 909 |  |
| Josse, 2011 [[7](#_ENREF_12)] (High dairy) | 15 | 16 | 0.8 | 0.8 | 520 ^2^ | 555 ^2^ |
| Josse, 2011 [[7](#_ENREF_12)] (Medium dairy) | 14 |  | 0.8 |  | 480 ^2^ |  |
| Lukaszuk, 2007 [[8](#_ENREF_45)] | NR | NR | NR | NR | NR | NR |
| Rosado, 2011 [[9](#_ENREF_51)] | NR | NR | NR | NR | NR | NR |
| Summerbell, 1998 [[10](#_ENREF_38)] | NR | NR | NR | NR | NR | NR |
| Tanaka, 2014 [[11](#_ENREF_41)] | 16.0 | 16.2 | NR | NR | 329 | 334 |
| Thomas, 2010 [[12](#_ENREF_22)] | 14.7 | 15.6 | 0.9 | 1.0 | 543 | 554 |
| Thomas, 2011 [[13](#_ENREF_44)] | 15.8 | 14.6 | 0.9 | 0.9 | 543 | 556 |
| Thompson, 2005 [[14](#_ENREF_40)] | NR | NR | NR | NR | NR | NR |
| Torres, 2010 [[15](#_ENREF_57)] | NR | NR | NR | NR | NR | NR |
| Van Loan, 2011 [[16](#_ENREF_52)] | NR | NR | NR | NR | NR | NR |
| Wagner, 2007 [[17](#_ENREF_53)] | NR | NR | NR | NR | 750 | 750 |
| Zemel, 2004 [[18](#_ENREF_58)] | NR | NR | NR | NR | NR | NR |
| Zemel, 2005a [[19](#_ENREF_56)] | NR | NR | NR | NR | NR | NR |
| Zemel, 2005b [20] | NR | NR | NR | NR | NR | NR |
| Zemel, 2009 [[21](#_ENREF_54)] | NR | NR | NR | NR | 488 | 496 |
| **Dairy Supplements** |  |  |  |  |  |  |
| Aldrich, 2011 [[22](#_ENREF_48)] | NR | NR | NR | NR | NR | NR |
| Anderson, 2007 [[23](#_ENREF_23)] | NR | NR | NR | NR | NR | NR |
| Frestedt, 2008 [[24](#_ENREF_47)] | 15.7 ^2^ | 16.6 ^2^ | 0.74 ^3^ | 0.76 ^3^ | 372 | 381 |
| Kasim-Karakas, 2009 [[25](#_ENREF_49)] | 20.7 | 17.0 | 0.78 ^3^ | 0.80 ^3^ | NR | NR |
| Longland, 2016 [[26](#_ENREF_42)] | NR | NR | NR | NR | NR | NR |
| Piccolo, 2015 [[27](#_ENREF_21)] | 17.8 | 18.4 | 0.80 ^3^ | 1.03 ^3^ | NR | NR |

CHO, carbohydrate; NR, not reported and not possible to calculate; % E, percentage of total energy intake; ^1^ First author, year of publication; ^2^ Percentage energy intake from protein, carbohydrate and fat calculated by converting average gram intakes to kJ by multiplying with Atwater factors (protein and carbohydrate = 16.736 kJ; fat = 37.656 kJ) and then calculating the percentage intake of the average total energy intake; ^3^ Calculated using average baseline body weight and average daily grams of protein intake.

**Table S2.** Daily nutrient intakes from intervention diets as reported by study participants.

| **Reference ^1^** | **Energy (kJ)** | | **Protein (% E)** | | **Protein (g/kg)** | | **Fat (% E)** | | **Carbohydrate (% E)** | | **Calcium (mg)** | |
| --- | --- | --- | --- | --- | --- | --- | --- | --- | --- | --- | --- | --- |
|  | **Dairy** | **Control** | **Dairy** | **Control** | **Dairy** | **Control** | **Dairy** | **Control** | **Dairy** | **Control** | **Dairy** | **Control** |
| **Dairy Food** |  |  |  |  |  |  |  |  |  |  |  |  |
| Anderson, 2005 [[1](#_ENREF_24)] | NR | NR | NR | NR | NR | NR | NR | NR | NR | NR | NR | NR |
| Bowen, 2005 [[2](#_ENREF_43)] (Energy restriction phase) | 5821 | 5936 | 31.7 | 29.8 | 1.2 ^3^ | 1.2 ^3^ | 26.0 | 29.5 | 38.8 | 37.6 | 2371 | 509 |
| Faghih, 2011 [[3](#_ENREF_55)] | 5430 | 5110 | 17.6 | 17.6 | 0.75 ^3^ | 0.70 ^3^ | 27.4 | 27.2 | 55.0 | 54.8 | 1302 | 496 |
| Gilbert, 2011 [[4](#_ENREF_20)] | 6513 | 6340 | 21.2 | 18.1 | 0.94 ^3^ | 0.80 ^3^ | 25.6 | 27.0 | 51.6 | 53.4 | 1523 | 733 |
| Harvey-Berino, 2005 [[5](#_ENREF_39)] (USA) | 6887 | 5991 | NR | NR | NR | NR | NR | NR | NR | NR | 1239 | 574 |
| Hawley, 2014 [[6](#_ENREF_50)] (High dairy + medium CHO) | 6689 | 6733 | 29 | 21 | 1.2 | 0.9 | 32 | 25 | 39 | 54 | 1398 | 721 |
| Hawley, 2014 [[6](#_ENREF_50)] (High dairy + high CHO) | 6791 |  | 31 |  | 1.3 |  | 22 |  | 47 |  | 1576 |  |
| Josse, 2011 [[7](#_ENREF_12)] (High dairy) | 6276 | 5523 | 28 | 16 | 1.33 | 0.72 | 31 | 28 | 41 | 56 | 1840 | 299 |
| Josse, 2011 [[7](#_ENREF_12)] (Medium dairy) | 5983 |  | 18 |  | 0.84 |  | 24 |  | 58 |  | 1200 |  |
| Lukaszuk, 2007 [[8](#_ENREF_45)] | 5028 | 5454 | 26.6 | 24.4 | 0.88 ^3^ | 0.78 ^3^ | 25.9 | 21.3 | 47.4 | 54.8 | 1189 | 1139 |
| Rosado, 2011 [[9](#_ENREF_51)] | NR | NR | NR | NR | NR | NR | NR | NR | NR | NR | NR | NR |
| Summerbell, 1998 [[10](#_ENREF_38)] | NR | NR | NR | NR | NR | NR | NR | NR | NR | NR | NR | NR |
| Tanaka, 2014 [[11](#_ENREF_41)] | 7715 | 7761 | 17.0 | 16.8 | NR | NR | 24.9 | 23.0 | 57.6 | 59.1 | 667 | 341 |
| Thomas, 2010 [[12](#_ENREF_22)] | 6548 | 6448 | 21.0 | 19.9 | 1.1 | 1.0 | 28.5 | 27.5 | 52.1 | 55.5 | 1311 | 445 |
| Thomas, 2011 [[13](#_ENREF_44)] | 6527 | 6464 | 21.9 | 18.8 | 1.1 | 1.0 | 26.5 | 29.7 | 54.4 | 53.1 | 931 | 790 |
| Thompson, 2005 [[14](#_ENREF_40)] | 6235 | 5971 | 21.5 | 18.8 | 0.81 ^3^ | 0.68 ^3^ | 24.6 | 26.3 | 53.6 | 54.5 | 1387 | 800 |
| Torres, 2010 [[15](#_ENREF_57)] | 7288 | 7146 | 21.5 | 20.9 | 1.1 ^3^ | 1.08 ^3^ | 28.3 | 27.8 | 50.2 | 41.4 | 1234 | 463 |
| Van Loan, 2011 [[16](#_ENREF_52)] | NR | NR | 16.6 ^4^ | 16.3 ^4^ | NR | NR | 35.7 ^4^ | 36.3 ^4^ | 49.5 ^4^ | 49.3 ^4^ | 1288 ^4^ | 435 ^4^ |
| Wagner, 2007 [[17](#_ENREF_53)] | 6179.8 | 5740.4 | NR | NR | NR | NR | NR | NR | NR | NR | 1514 | 788 |
| Zemel, 2004 [[18](#_ENREF_58)] | 5732 | 5477 | 18 | 17 | 0.61 ^3^ | 0.54 ^3^ | 31 | 32 | 51 | 52 | 1137 | 430 |
| Zemel, 2005a [[19](#_ENREF_56)] | 6238 | 5347 | 18 | 17 | NR | NR | 31 | 30 | 51 | 53 | NR | NR |
| Zemel, 2005b [[20](#_ENREF_46)] | 6012 | 5451 | 18 | 18 | NR | NR | 30 | 30 | 52 | 52 | 1077 | 495 |
| Zemel, 2009 [[21](#_ENREF_54)] | 6356 | 5610 | 18.7 ^2^ | 17.9 ^2^ | 0.89 ^3^ | 0.75 ^3^ | 29 ^2^ | 28.2 ^2^ | 50.3 ^2^ | 53.1 ^2^ | NR | NR |
| **Dairy Supplements** |  |  |  |  |  |  |  |  |  |  |  |  |
| Aldrich, 2011 [[22](#_ENREF_48)] | 6698 | 6699 | 31 ^2^ | 15.8 ^2^ | 1.46 ^3^ | 0.78 ^3^ | 30.8 ^2^ | 30.9 ^2^ | 41.7 ^2^ | 56.3 ^2^ | 1536 | 1316 |
| Anderson, 2007 [[23](#_ENREF_23)] | NR | NR | NR | NR | NR | NR | NR | NR | NR | NR | NR | NR |
| Frestedt, 2008 [[24](#_ENREF_47)] | 5780 | 5789 | 16.5 ^2^ | 16.8 ^2^ | 0.81 ^3^ | 0.61 ^3^ | 31.9 ^2^ | 30.6 ^2^ | 51.5 ^2^ | 52.6 ^2^ | 275 | 317 |
| Kasim-Karakas, 2009 [25] | 5770 | 5657 | 33.7 | 16.6 | 1.07 ^3^ | 0.61 ^3^ | 26.2 | 25.9 | 39.5 | 56.7 | NR | NR |
| Longland, 2016 [[26](#_ENREF_42)] | 10093 ^4^ | 9555 ^4^ | 35 ^4^ | 15 ^4^ | 2.4 ^4^ | 1.2 ^4^ | 15 ^4^ | 35 ^4^ | 50 ^4^ | 50 ^4^ | NR | NR |
| Piccolo, 2015 [[27](#_ENREF_21)] | 5302 | 5649 | 20 | 20.8 | 0.68 ^3^ | 0.78 ^3^ | 31.9 | 32.2 | 48.1 | 47.0 | NR | NR |

NR, not reported and not possible to calculate; % E, percentage of total energy intake. To convert kJ to kcal: 1 kcal = 4.184 kJ; ^1^ First author, year of publication; ^2^ Percentage energy intake from protein, carbohydrate or fat calculated by converting average gram intakes to kJ by multiplying with Atwater factors (protein and carbohydrate = 16.736 kJ; fat = 37.656 kJ) and then calculating the percentage intake of the average total energy intake; ^3^ Calculated by dividing average daily grams of protein intake (reported or calculated from percentage energy protein) by average baseline body weight; ^4^ Controlled feeding trial (all food provided).

**Table S3.** Quality appraisal of included studies.

| **Reference ^1^** | **In-/Exclusion Criteria** | **Group Allocation** | | | | **Blinding** | | **Attrition** | | **Intervention** | | **Health Effect** | **Statistical Analysis** | | **Confounders** | **Total** | **Potential Confounders Not Considered by the Authors** |
| --- | --- | --- | --- | --- | --- | --- | --- | --- | --- | --- | --- | --- | --- | --- | --- | --- | --- |
|  |  | **Randomized** | **Method Reported** | **Method Appropriate** | **Allocation Concealed** | **Participants** | **Researchers** | **Numerically Reported** | **Reasons Provided** | **Type Described** | **Amount Described** | **Methodology Reported** | **Between-Groups** | **Intention-to-Treat** |  |  |  |
| **Dairy Food** |  |  |  |  |  |  |  |  |  |  |  |  |  |  |  |  |  |
| Anderson, 2005 [[1](#_ENREF_24)] | 1 | 1 | 1 | 1 | 1 | 0 | 0 | 1 | 1 | 1 | 1 | 0 | 1 | 1 | 0 | 11 | Sex distributions across groups not equal (more men in milk group). Quantity of meal replacements not matched across groups (2× milk vs. 5× soy/day). Attrition bias unclear; reasons for attrition not reported across groups. Nutrient intakes not reported; can’t determine whether similar across groups or whether energy targets achieved. |
| Bowen, 2005 [[2](#_ENREF_43)] | 1 | 1 | 0 | 0 | 0 | 0 | 0 | 1 | 1 | 1 | 1 | 1 | 1 | 0 | 0 | 8 | Attrition bias unclear; number and reasons for dropouts not reported across groups. Females in dairy group significantly greater fat mass at baseline than control group. |
| Faghih, 2011 [[3](#_ENREF_55)] | 1 | 1 | 0 | 0 | 0 | 0 | 0 | 1 | 0 | 1 | 1 | 1 | 1 | 0 | 0 | 7 | Attrition bias unclear—reasons for dropout not reported. |

| Gilbert, 2011 [[4](#_ENREF_20)] | 1 | 1 | 0 | 0 | 0 | 1 | 0 | 1 | 1 | 1 | 1 | 1 | 1 | 0 | 1 | 10 | None identified |
| --- | --- | --- | --- | --- | --- | --- | --- | --- | --- | --- | --- | --- | --- | --- | --- | --- | --- |
| Harvey-Berino, 2005 [[5](#_ENREF_39)] | 1 | 1 | 0 | 0 | 0 | 0 | 0 | 1 | 1 | 1 | 1 | 1 | 1 | 1 | 0 | 9 | Attrition bias unclear; reasons for attrition not reported across groups. Greater attrition in control group (8 vs. 2). Macronutrient intake not reported to confirm no differences between groups. |
| Hawley, 2014 [[6](#_ENREF_50)] | 1 | 1 | 1 | 0 | 0 | 0 | 0 | 1 | 1 | 1 | 1 | 1 | 1 | 0 | 0 | 9 | Attrition bias; non-completers had greater baseline body weight and trunk fat vs. completers. |
| Josse, 2011 [[7](#_ENREF_12)] | 1 | 1 | 1 | 1 | 0 | 0 | 1 | 1 | 1 | 1 | 1 | 1 | 1 | 1 | 1 | 13 | None identified |
| Lukaszuk, 2007 [[8](#_ENREF_45)] | 1 | 1 | 1 | 1 | 0 | 0 | 0 | 1 | 1 | 1 | 1 | 1 | 1 | 0 | 0 | 10 | Soy group greater BMI and body fat although not significant (likely due to small sample size). Soy group higher CHO intake than dairy group. |
| Rosado, 2011 [[9](#_ENREF_51)] | 1 | 1 | 1 | 1 | 0 | 0 | 0 | 1 | 1 | 1 | 1 | 1 | 1 | 1 | 0 | 11 | Attrition bias; reasons for withdrawal across groups not equal. Attrition 2x greater in control group. More participants in control group not adhering to treatment. No dietary data reported to determine whether groups differed. |
| Summerbell, 1998 [[10](#_ENREF_38)] | 1 | 1 | 1 | 1 | 1 | 0 | 1 | 1 | 1 | 1 | 1 | 1 | 1 | 1 | 0 | 13 | Very little data provided to determine confounding factors. Dairy diet consisted of milk only for the 16 week study period. |
| Tanaka, 2014 [[11](#_ENREF_41)] | 1 | 1 | 1 | 1 | 1 | 0 | 0 | 1 | 0 | 1 | 1 | 1 | 1 | 1 | 1 | 12 | None identified |
| Thomas, 2010 [[12](#_ENREF_22)] | 1 | 1 | 1 | 1 | 1 | 0 | 0 | 1 | 1 | 1 | 1 | 1 | 1 | 0 | 1 | 12 | None identified |
| Thomas, 2011 [[18](#_ENREF_44)] | 1 | 1 | 0 | 0 | 0 | 0 | 1 | 1 | 1 | 1 | 1 | 1 | 1 | 0 | 1 | 10 | None identified |
| Thompson, 2005 [[14](#_ENREF_40)] | 1 | 1 | 1 | 1 | 1 | 0 | 0 | 1 | 1 | 1 | 1 | 1 | 1 | 1 | 1 | 13 | None identified |
| Torres, 2010 [15] | 1 | 1 | 1 | 1 | 0 | 0 | 0 | 1 | 1 | 1 | 1 | 1 | 1 | 0 | 1 | 11 | None identified |
| Van Loan, 2011 [[16](#_ENREF_52)] | 1 | 1 | 0 | 0 | 0 | 0 | 0 | 1 | 0 | 1 | 1 | 1 | 1 | 0 | 0 | 7 | Attrition bias unclear; reasons for attrition not provided. Baseline characteristics reported for total group; can’t determine whether groups similar at baseline. |
| Wagner, 2007 [[17](#_ENREF_53)] | 1 | 1 | 1 | 1 | 1 | 0 | 0 | 0 | 0 | 1 | 1 | 1 | 1 | 0 | 0 | 9 | Nutrient intake reporting incomplete. Unclear whether nutrient intakes differed between groups. |
| Zemel, 2004 [[18](#_ENREF_58)] | 1 | 1 | 0 | 0 | 0 | 0 | 0 | 1 | 0 | 1 | 1 | 1 | 1 | 0 | 1 | 8 | None identified |
| Zemel, 2005a [[19](#_ENREF_56)] | 1 | 1 | 0 | 0 | 0 | 0 | 0 | 1 | 1 | 1 | 1 | 1 | 1 | 0 | 1 | 9 | None identified |
| Zemel, 2005b [[20](#_ENREF_46)] | 1 | 1 | 0 | 0 | 0 | 0 | 0 | 1 | 1 | 1 | 1 | 1 | 1 | 0 | 1 | 9 | None identified |
| Zemel, 2009 [[21](#_ENREF_54)] | 1 | 1 | 0 | 0 | 0 | 0 | 0 | 1 | 1 | 1 | 1 | 1 | 1 | 1 | 0 | 9 | Attrition bias unclear; reasons for attrition not reported. |
| **Dairy Supplements** |  |  |  |  |  |  |  |  |  |  |  |  |  |  |  |  |  |
| Aldrich, 2011 [[22](#_ENREF_48)] | 1 | 1 | 0 | 0 | 0 | 0 | 0 | 1 | 1 | 1 | 1 | 1 | 1 | 0 | 0 | 8 | Calcium supplement added to control group to balance Calcium intake. Whey group consumed 0.5 serving of dairy/daymore than control group. |
| Anderson, 2007 [[23](#_ENREF_23)] | 1 | 1 | 0 | 0 | 0 | 1 | 0 | 1 | 1 | 1 | 1 | 1 | 1 | 1 | 0 | 10 | No dietary intake information reported to determine whether groups differed. |
| Frestedt, 2008 [[24](#_ENREF_47)] | 1 | 1 | 0 | 0 | 0 | 0 | 0 | 1 | 1 | 1 | 1 | 1 | 1 | 0 | 0 | 8 | Very high attrition rate (56%) over 12 weeks. |
| Kasim-Karakas, 2009 [[25](#_ENREF_49)] | 1 | 1 | 1 | 1 | 0 | 0 | 1 | 1 | 1 | 1 | 1 | 1 | 1 | 0 | 0 | 11 | Calcium added to control treatment, and intakes not measured to confirm. Compliance not assessed. Fat intake in control group lower at baseline. |
| Longland, 2016 [26] | 1 | 1 | 1 | 1 | 0 | 1 | 0 | 1 | 1 | 1 | 1 | 1 | 1 | 1 | 0 | 12 | Fat intake during intervention significantly higher in control group (0.9 vs. 0.4 g/kg, *p* = 0.01) |
| Piccolo, 2015 [27] | 1 | 1 | 0 | 0 | 0 | 1 | 1 | 1 | 0 | 1 | 1 | 1 | 1 | 0 | 0 | 9 | Attrition bias unclear; reasons for attrition not provided; unequal numbers in intervention and control groups with no reasons provided. |

BMI, Body mass index; CHO, Carbohydrate; ^1^ First author, year of publication.


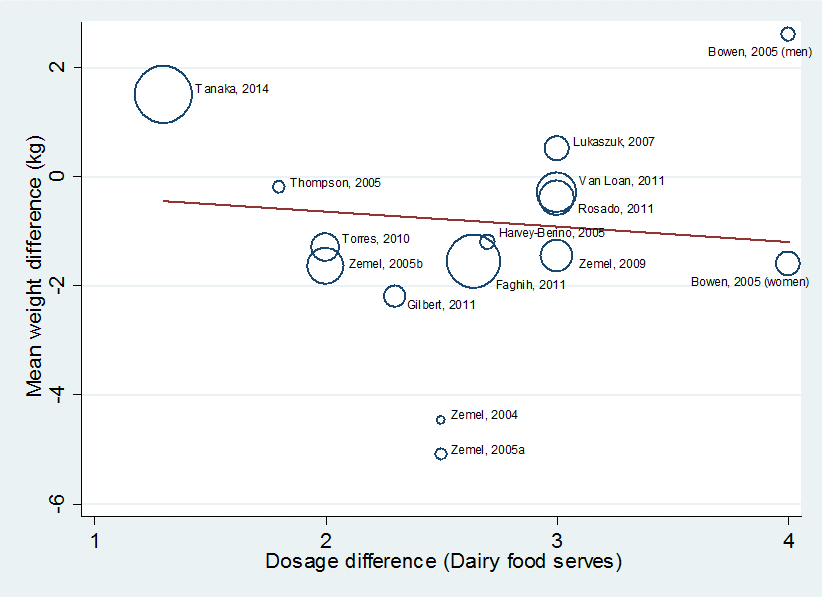


**Figure S1.** Mean body weight difference (kg) between high dairy and control groups by difference in daily serves of dairy food intake for trials using whole dairy foods (*n* = 15). The size of the circle is proportional to the inverse of the within-study variance of effect size.

| 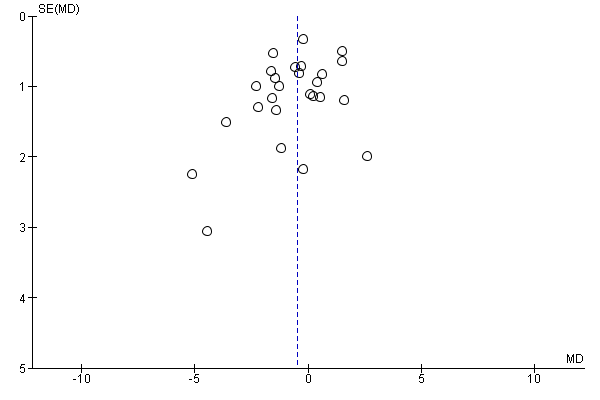 | 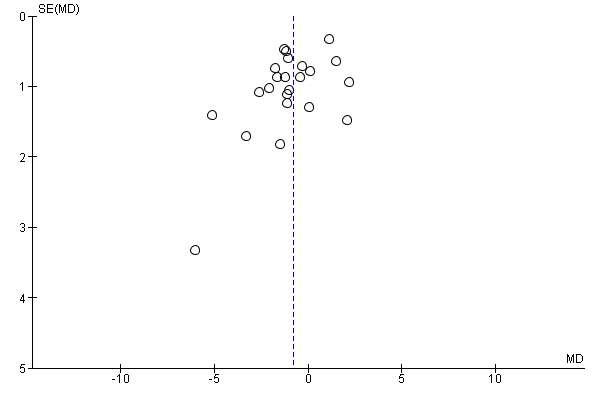 |
| --- | --- |
| (**A**) | (**B**) |
| 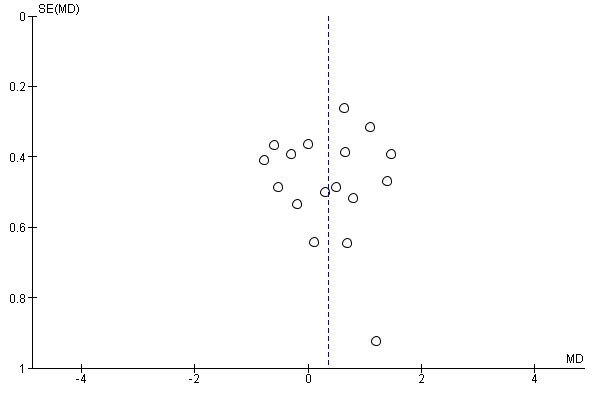 | |
| (**C**) | |

**Figure S2.** Funnel plot for (**A**) Body weight (kg); (**B**) Body fat mass (kg); (**C**) Body lean mass (kg); SE, standard error; MD, mean weighted difference between high dairy and control groups.

**References**

1. Anderson, J.W.; Hoie, L.H. Weight loss and lipid changes with low-energy diets: Comparator study of milk-based versus soy-based liquid meal replacement interventions. *J. Am. Coll. Nutr*. **2005**, *24*, 210–216.
2. Bowen, J.; Noakes, M.; Clifton, P.M. Effect of calcium and dairy foods in high protein, energy-restricted diets on weight loss and metabolic parameters in overweight adults. *Int. J. Obes. (Lond.)* **2005**, *29*, 957–965.
3. Faghih, S.; Abadi, A.R.; Hedayati, M.; Kimiagar, S.M. Comparison of the effects of cows’ milk, fortified soy milk, and calcium supplement on weight and fat loss in premenopausal overweight and obese women. *Nutr. Metab. Cardiovasc. Dis*. **2011**, *21*, 499–503.
4. Gilbert, J.A.; Joanisse, D.R.; Chaput, J.P.; Miegueu, P.; Cianflone, K.; Almeras, N.; Tremblay, A. Milk supplementation facilitates appetite control in obese women during weight loss: A randomised, single-blind, placebo-controlled trial. *Br. J. Nutr*. **2011**, *105*, 133–143.
5. Harvey-Berino, J.; Gold, B.C.; Lauber, R.; Starinski, A. The impact of calcium and dairy product consumption on weight loss. *Obes. Res*. **2005**, *13*, 1720–1726.
6. Hawley, J.A. *Effect of Dairy-Based High Protein, Variable-Carbohydrate Diets and Exercise on Muscle Maintenance and Movement*; Dairy Health Nutrition Consortium: Werribee, Australia, 2014.
7. Josse, A.R.; Atkinson, S.A.; Tarnopolsky, M.A.; Phillips, S.M. Increased consumption of dairy foods and protein during diet- and exercise-induced weight loss promotes fat mass loss and lean mass gain in overweight and obese premenopausal women. *J. Nutr*. **2011**, *141*, 1626–1634.
8. Lukaszuk, J.M.; Luebbers, P.; Gordon, B.A. Preliminary study: Soy milk as effective as skim milk in promoting weight loss. *J. Am. Diet. Assoc*. **2007**, *107*, 1811–1814.
9. Rosado, J.L.; Garcia, O.P.; Ronquillo, D.; Hervert-Hernandez, D.; Caamano, M.D.C.; Martinez, G.; Gutiérrez, J.; García, S. Intake of milk with added micronutrients increases the effectiveness of an energy-restricted diet to reduce body weight: A randomized controlled clinical trial in Mexican women. *J. Am. Diet. Assoc*. **2011**, *111*, 1507–1516.
10. Summerbell, C.D.; Watts, C.; Higgins, J.P.T.; Garrow, J.S. Randomised controlled trial of novel, simple, and well supervised weight reducing diets in outpatients. *Br. Med. J*. **1998**, *317*, 1487–1489.
11. Tanaka, S.; Uenishi, K.; Ishida, H.; Takami, Y.; Hosoi, T.; Kadowaki, T.; Orimo, H.; Ohashi, Y. A randomized intervention trial of 24-wk dairy consumption on waist circumference, blood pressure, and fasting blood sugar and lipids in Japanese men with metabolic syndrome. *J. Nutr. Sci. Vitaminol. (Tokyo)* **2014**, *60*, 305–312.
12. Thomas, D.T.; Wideman, L.; Lovelady, C.A. Effects of calcium and resistance exercise on body composition in overweight premenopausal women. *J. Am. Coll. Nutr*. **2010**, *29*, 604–611.
13. Thomas, D.T.; Wideman, L.; Lovelady, C.A. Effects of a dairy supplement and resistance training on lean mass and insulin-like growth factor in women. *Int. J. Sport Nutr. Exerc. Metab.* **2011**, *21*, 181–188.
14. Thompson, W.G.; Rostad Holdman, N.; Janzow, D.J.; Slezak, J.M.; Morris, K.L.; Zemel, M.B. Effect of energy-reduced diets high in dairy products and fiber on weight loss in obese adults. *Obes. Res*. **2005**, *13*, 1344–1353.
15. Torres, M.R.; Francischetti, E.A.; Genelhu, V.; Sanjuliani, A.F. Effect of a high-calcium energy-reduced diet on abdominal obesity and cardiometabolic risk factors in obese Brazilian subjects. *Int. J. Clin. Pract*. **2010**, *64*, 1076–1083.
16. Van Loan, M.D.; Keim, N.L.; Adams, S.H.; Souza, E.; Woodhouse, L.R.; Thomas, A.; Witbracht, M.; Gertz, E.R.; Piccolo, B.; Bremer, A.A.; et al. Dairy foods in a moderate energy restricted diet do not enhance central fat, weight, and intra-abdominal adipose tissue losses nor reduce adipocyte size or inflammatory markers in overweight and obese adults: A controlled feeding study. *J. Obes*. **2011**, *2011*, 989657.
17. Wagner, G.; Kindrick, S.; Hertzler, S.; DiSilvestro, R.A. Effects of various forms of calcium on body weight and bone turnover markers in women participating in a weight loss program. *J. Am. Coll. Nutr*. **2007**, *26*, 456–461.
18. Zemel, M.B.; Thompson, W.; Milstead, A.; Morris, K.; Campbell, P. Calcium and dairy acceleration of weight and fat loss during energy restriction in obese adults. *Obes. Res*. **2004**, *12*, 582–590.
19. Zemel, M.B.; Richards, J.; Milstead, A.; Campbell, P. Effects of calcium and dairy on body composition and weight loss in African-American adults. *Obes. Res*. **2005**, *13*, 1218–1225.
20. Zemel, M.B.; Richards, J.; Mathis, S.; Milstead, A.; Gebhardt, L.; Silva, E. Dairy augmentation of total and central fat loss in obese subjects. *Int. J. Obes. (Lond.)* **2005**, *29*, 391–397.
21. Zemel, M.B.; Teegarden, D.; Loan, M.V.; Schoeller, D.A.; Matkovic, V.; Lyle, R.M.; Craig, B.A. Dairy-rich diets augment fat loss on an energy-restricted diet: A multicenter trial. *Nutrients* **2009**, *1*, 83–100.
22. Aldrich, N.D.; Reicks, M.M.; Sibley, S.D.; Redmon, J.B.; Thomas, W.; Raatz, S.K. Varying protein source and quantity do not significantly improve weight loss, fat loss, or satiety in reduced energy diets among midlife adults. *Nutr. Res.* **2011**, *31*, 104–112.
23. Anderson, J.W.; Fuller, J.; Patterson, K.; Blair, R.; Tabor, A. Soy compared to casein meal replacement shakes with energy-restricted diets for obese women: Randomized controlled trial. *Metabolism* **2007**, *56*, 280–288.
24. Frestedt, J.L.; Zenk, J.L.; Kuskowski, M.A.; Ward, L.S.; Bastian, E.D. A whey-protein supplement increases fat loss and spares lean muscle in obese subjects: A randomized human clinical study. *Nutr. Metab.* **2008**, *5*, 8.
25. Kasim-Karakas, S.E.; Almario, R.U.; Cunningham, W. Effects of protein versus simple sugar intake on weight loss in polycystic ovary syndrome (according to the National Institutes of Health criteria). *Fertil. Steril*. **2009**, *92*, 262–270.
26. Longland, T.M.; Oikawa, S.Y.; Mitchell, C.J.; Devries, M.C.; Phillips, S.M. Higher compared with lower dietary protein during an energy deficit combined with intense exercise promotes greater lean mass gain and fat mass loss: A randomized trial. *Am. J. Clin. Nutr*. **2016**, *103*, 738–746.
27. Piccolo, B.D.; Comerford, K.B.; Karakas, S.E.; Knotts, T.A.; Fiehn, O.; Adams, S.H. Whey protein supplementation does not alter plasma branched-chained amino acid profiles but results in unique metabolomics patterns in obese women enrolled in an 8-week weight loss trial. *J. Nutr*. **2015**, *145*, 691–700.
